# ATRX is a regulator of therapy induced senescence in human cells

**DOI:** 10.1038/s41467-017-00540-5

**Published:** 2017-08-30

**Authors:** Marta Kovatcheva, Will Liao, Mary E. Klein, Nicolas Robine, Heather Geiger, Aimee M. Crago, Mark A. Dickson, William D. Tap, Samuel Singer, Andrew Koff

**Affiliations:** 1 The Louis V. Gerstner Graduate School of Biomedical Sciences, Sloan Kettering Institute, Memorial Sloan Kettering Cancer Center, New York, 10065 USA; 2 Program in Molecular Biology, Memorial Sloan Kettering Cancer Center, New York, 10065 USA; 3 The New York Genome Center, New York, 10013 USA; 4 Department of Surgery, Memorial Sloan Kettering Cancer Center, New York, 10065 USA; 5 Department of Medicine, Weill College of Medicine, Cornell University, New York, 10065 USA; 6 Department of Medicine, Memorial Sloan Kettering Cancer Center, New York, 10065 USA

## Abstract

Senescence is a state of stable cell cycle exit with important implications for development and disease. Here, we demonstrate that the chromatin remodeling enzyme ATRX is required for therapy-induced senescence. ATRX accumulates in nuclear foci and is required for therapy-induced senescence in multiple types of transformed cells exposed to either DNA damaging agents or CDK4 inhibitors. Mobilization into foci depends on the ability of ATRX to interact with H3K9me3 histone and HP1. Foci form soon after cells exit the cell cycle, before other hallmarks of senescence appear. Eliminating ATRX in senescent cells destabilizes the senescence-associated heterochromatic foci. Additionally, ATRX binds to and suppresses expression from the *HRAS* locus; repression of *HRAS* is sufficient to promote the transition of quiescent cells into senescence and preventing repression blocks progression into senescence. Thus ATRX is a critical regulator of therapy-induced senescence and acts in multiple ways to drive cells into this state.

## Abstract (web summary)

Therapy induced senescence (TIS) is a growth suppressive program activated by cytostatic agents in some cancer cells. Here the authors show that the chromatin remodeling enzyme ATRX is a regulator of TIS and drives cells into this state via multiple mechanisms.

## Introduction

Quiescent cells have withdrawn from the mitotic cycle and retain the capacity to return. Senescent cells have withdrawn from the mitotic cycle and are refractory to signals that could stimulate their return. They can also elaborate a cytokine expression program leading to “sterile” inflammation in the surrounding area known as the senescence-associated secretory phenotype (SASP)^1^. The replicative proficiency of cells that have exited the cell cycle has important consequences for tumor suppression, aging, development and disease^2–5^. For example, stem cell pools are actively maintained in quiescence^6–8^. Additionally, the inflammatory program induced in senescent cells can contribute to some of the pathologies associated with aging^2, 9, 10^.

Cellular senescence can be triggered by various stresses. The best understood molecular paradigms of cellular senescence are replicative senescence (associated with telomere loss leading to a chronic DNA damage response in primary cells), oncogene-induced senescence (OIS, associated with hyper-replicative stress leading to a chronic DNA damage response, genome instability, and accumulation of p16 and p53 in primary cells), and Pten-loss induced cellular senescence (PICS, associated with SKP2 dependent regulation of the CDK inhibitor p27 but not with hyper-replicative stress or the accumulation of p16 and p53 in primary prostate epithelial cells)^11^. The most poorly understood, but practically important type of cellular senescence, is therapy-induced senescence (TIS), which is a growth suppressive program activated by cytostatic agents in some cancer cells (reviewed in refs^12, 13^).

Regardless of the mode of induction, two key features of all senescent cells are that they elaborate a cytokine expression program leading to inflammation (SASP) and there is an increase in facultative heterochromatin known as the senescence-associated heterochromatic foci (SAHF). Collectively these conspire to prevent the cells from returning to the cell cycle once the inducing signal is removed. NFκB, GATA6 and BRD4 transcriptional networks sculpt the inflammatory response^14–16^. Senescent cells are identified by a number of associated hallmarks including failure to replicate DNA, elaboration of the SASP, accumulation of SAHF (defined as an increase in focal localization of the HP1 family of proteins) and the accumulation of senescence-associated β-galactosidase (SA-β-gal) activity. Most importantly, these cells are unable to return to cell cycle once the inducer has been removed. Typically, some but not all such hallmarks accumulate leading to some controversy over what is a senescent cell^17^.

The mechanism of SAHF formation has been extensively reviewed^18–20^. Although SAHF are not observed in all contexts in which senescence occurs, when they do form they are required for senescence^18, 21–25^. SAHF are identified by focal chromatin deposition of Rb, the histone variant macroH2A (mH2A), the HP1 family of proteins, the high mobility group proteins (HMGA), the accumulation of proteolytically processed histone H3.3, and the accumulation of H3K9me3 histone^18, 21, 22, 25–28^.

The assembly of SAHF begins with the transit of both HIRA and HP1 proteins to PML nuclear bodies (PML-NBs). There, HP1γ may be phosphorylated, which is required for its deposition into SAHF. HIRA associates with the histone chaperone ASF1 to deposit H3.3-containing nucleosome complexes and facilitate chromatin condensation, likely due to increased nucleosome density. Histone methyltransferases then catalyze the K9me3 modification of these nucleosomes, which allows recruitment of HP1 proteins. mH2A is incorporated into SAHF around the same time as HP1. It is unclear when HMGA is incorporated into SAHF, although it is presumably an early event^18, 21, 22^.

ATRX is another chromatin remodeling enzyme that can facilitate replication independent histone H3.3 deposition^29^. In cycling cells, ATRX, in association with the histone H3 chaperone DAXX, maintains the constitutive heterochromatin at telomeric and pericentromeric regions^30^. ATRX can also regulate facultative heterochromatin. ATRX can repress imprinted genes in mouse embryonic stem cells^31, 32^, and participates in the process of X-chromosome inactivation^33^. On the other hand, ATRX can stimulate gene expression by preventing the deposition of mH2A at the α-globin locus^34^, and has been shown to indirectly regulate the turnover of MDM2 after cells exit the cell cycle following treatment with CDK4 inhibitors (CDK4i)^35^. ATRX also has roles independent of transcription. For example, ATRX is required for DNA replication^36–38^ and it can localize to sites of DNA damage^39^.

Interestingly, ATRX can interact with a number of proteins involved in the formation of the SAHF, such as PML^40, 41^, as well as structural proteins of the SAHF, such as the HP1 family of proteins^42^ and H3K9me3 histone^42, 43^. Thus, we set out to determine whether ATRX played a role in TIS. Here we report that ATRX is a critical regulator of TIS induced by multiple treatments. ATRX is recruited into nuclear foci soon after a senescence inducer is applied, and cells lacking ATRX fail to become irreversibly arrested but still undergo cell cycle exit. Recruitment into foci depends on the ability of ATRX to interact with H3K9me3-modified histones and HP1 proteins. Reducing ATRX after senescence destabilizes the SAHF. ATRX also binds to and represses expression from the *HRAS* locus in cells that are undergoing senescence, and manipulating *HRAS* expression can alter the outcome of the inducer. Thus, ATRX is a critical regulator promoting the transition of quiescent cells into senescence.

## Results

### ATRX is a novel regulator of senescence

**Fig. 1:**
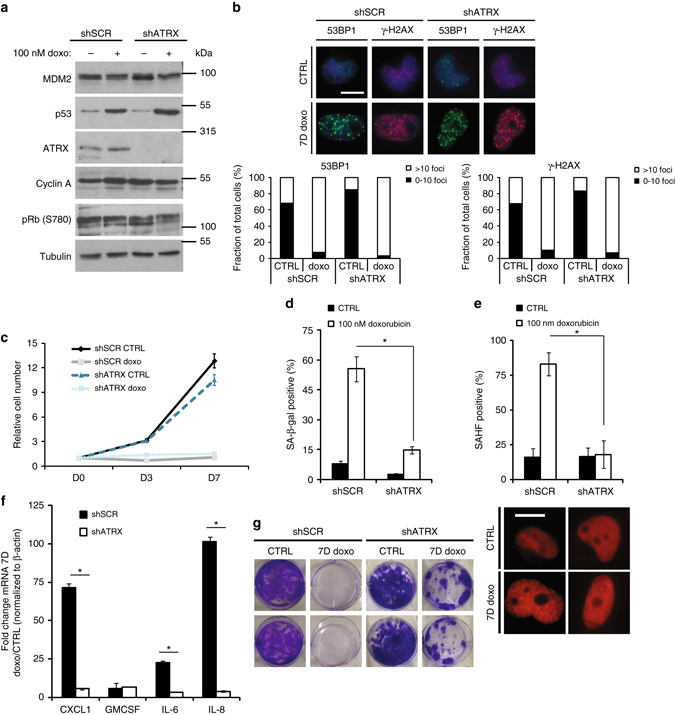
ATRX is necessary for doxorubicin-induced senescence. LS8817 cells were transduced with either a scrambled (shSCR) or ATRX-specific (shATRX) lentiviral knockdown vector and subsequently treated with 100 nM doxorubicin for 7 days. **a** Extracts were made from the cells indicated above each lane and the expression of proteins determined by immunoblot as indicated on the *left* of each panel. ATRX was detected with the D-5 antibody. **b** Cells were treated as described on the *left* and stained with the antibodies indicated on *top* of each panel. Representative images are shown (*top*) and quantification of the foci is plotted (*bottom*). **c** The indicated cells were plated on day 0 and either treated with 100 nM doxorubicin (doxo) or left untreated (CTRL). Cell number was counted at the indicated days and the relative number of cells is plotted. **d**, **e** The accumulation of SA-β-gal-positive cells (**d**) or SAHF-positive cells (**e**) for each individual treatment condition was measured. Representative SAHF images are shown (*below*). **f** The accumulation of four of the liposarcoma SASP transcripts was measured by qPCR under each indicated condition and their induction in doxorubicin treated cells relative to that in untreated cells is plotted and compared. **g** The cells were treated with 100 nM doxorubicin (7D doxo) or left untreated (CTRL) for 7 days. Cells were then collected, counted and replated at low density in the absence of drug. Clonogenic growth was measured by crystal violet staining 3 weeks later. Representative images from two independent experiments are shown. Colony number is not quantified as individual colonies could not be discerned. All data are plotted as mean ± SEM from at least three independent experiments. *Asterisk* indicates *p* < 0.05 using a two-sided Student’s *t-*test. All images were taken at the same magnification. The *scale bar* is 20 microns. See also Supplementary Fig. 10 for uncropped blots

Unlike CDK4 inhibition, doxorubicin causes DNA damage and induces senescence in an MDM2-independent but p53-dependent manner in G2/M arrested cells^44^. In order to determine whether ATRX played a role in doxorubicin-induced senescence we generated paired isogenic LS8817 cells: in one, LS8817^shATRX^, we reduced ATRX by lentiviral transduction of a vector expressing an shRNA (shX590), and in the other, LS8817^scr^, we expressed an shRNA with a scrambled sequence. LS8817 cells were derived from well-differentiated and dedifferentiated liposarcoma (WD/DDLS). Reducing ATRX in these cells did not affect the expression of cyclin A or phospho-Rb, nor did it affect the accumulation of p53 (Fig. 1a), the accumulation of 53BP1 or γH2Ax foci (Fig. 1b), or affect cell number (Fig. 1c) following treatment with doxorubicin. However, it did affect the accumulation of SA-β-gal-positive cells (Fig. 1d), SAHF-positive cells (Fig. 1e), the accumulation of three of the four mRNAs (CXCL1, GM-CSF, IL-6, and IL-8) that increase as part of the SASP in LS8817 cells (Fig. 1f and see Methods section), and the ability of the cells to return to the cell cycle following drug removal and replating (Fig. 1g). Similarly, cyclin A was not downregulated following doxorubicin treatment (Supplementary Fig. 1B), and the accumulation of SA-β-gal (Supplementary Fig. 1C), SAHF (Supplementary Fig. 1D), and SASP mRNAs (Supplementary Fig. 1E) were decreased in another pair of isogenic LS8817 cell lines created with a different previously validated ATRX shRNA (shX588)^35^.

Loss of MDM2 can also induce senescence in transformed cells^35^. Using the isogenic LS8817 cells, and another genetically distinct WD/DDLS cell line, LS8313, in which we generated isogenic pairs, reducing MDM2 led to accumulation of cells in G1 with reduced levels of cyclin A and phospho-Rb (Supplementary Fig. 2A). However, accumulation of SA-β-gal was attenuated in the ATRX-deficient cells (Supplementary Fig. 2B). Thus, ATRX was required for senescence induced by either doxorubicin or by MDM2 loss.

We then asked whether reciprocally restoring full-length ATRX expression in Rb-positive U2OS cells would increase the number of cells that undergo senescence when treated with CDK4i. U2OS cells have a deletion encompassing exons 2–19 of ATRX^45^. Both parental and ATRX expressing cells exited the cell cycle within two days of CDK4 inhibition as measured by decreased accumulation of phosphorylated Rb and cyclin A (Supplementary Fig. 3A). However after seven days of drug treatment, the number of ATRX expressing cells that were SA-β-gal (Supplementary Fig. 3B) or SAHF-positive (Supplementary Fig. 3C) increased compared to the parental cells. Furthermore, the ability of the ATRX expressing cells to return to the cell cycle following drug removal and replating was compromised (Supplementary Fig. 3D). For the month that we maintained such cells, ATRX expression did not significantly alter their proliferation in unperturbed culture, nor did it affect their viability after CDK4i induced cell cycle exit.

Restoration of ATRX expression in U2OS cells had been shown to diminish the long telomeres associated with the ALT phenotype (ref. 46). However, using a telomere restriction fragment (TRF) assay similar to the one used by Clynes and colleagues we were unable to score such a reversal (Supplementary Fig. 3E). This might be due to experimental differences in the levels of ATRX expression, as we selected for low expressing cells and they used a doxocycline inducible system. Thus we suspected that ATRXs function in senescence may extend beyond its well known ability to regulate telomere length and biology. Regardless it was clear that ATRX was necessary for multiple types of TIS. DNA damage, known to lead to G2 cell accumulation and act in a p53-dependent manner and CDK4 inhibition, known to lead to G1 accumulation and act in a p53/Ink4-independent manner.

### The number of nuclear ATRX foci increases during TIS

ATRX is a SWI/SNF family chromatin remodeling protein that can interact with the chromatin through a diverse collection of mechanisms. It can associate with the histone H3 chaperone DAXX, the structural protein HP1, K9-trimethylated and K4 non-methylated modifications on histone H3 (H3K9me3 and H3K4me0), and mH2A. mH2A, HP1 and H3K9me3 are all components of the SAHF^18, 21, 22, 25–27^. Thus we looked to see if the cytological appearance of ATRX changes in senescent cells, and if ATRX colocalized with the SAHF.

**Fig. 2:**
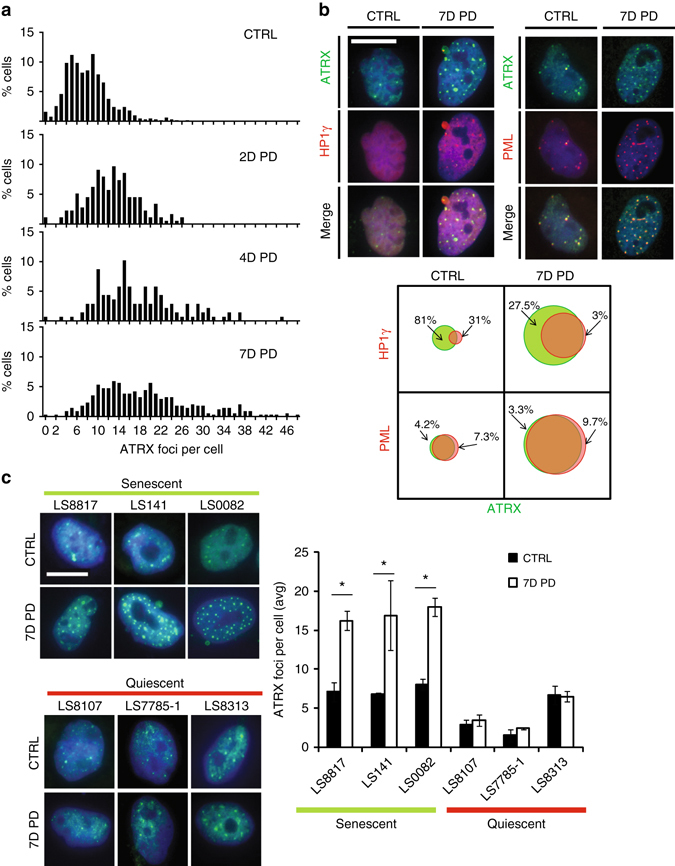
ATRX accumulates in nuclear foci in senescent cells that colocalize with HP1γ SAHF. **a** LS8817 cells were treated with PD0332991 for the indicated number of days, fixed and subsequently stained with ATRX antibodies. The number of ATRX foci in each cell (>150 cells per graph) were counted and plotted. The percentage of cells with a number of foci is indicated on the *y*-axis. **b** LS8817 cells were treated with PD0332991 for 7 days and the co-localization of HP1γ (*left*) or PML foci (*right*) with ATRX foci determined by immunofluorescence. Representative images are shown. The fraction of ATRX foci co-localized with either HP1γ or PML foci was quantified in control and treated cells. *Circles* in the Venn diagrams are drawn to scale relative to the number of the indicated foci in each condition. **c** The indicated liposarcoma cell lines were treated with PD0332991 for 7 days as previously described^35^. The outcome of this treatment vis a vis quiescence or senescence is indicated. ATRX foci were detected by immunofluorescence and the average number of foci per cell is plotted (*right*). Representative images are shown on the *left*. All data are represented as mean ± SEM from at least 3 independent experiments. *Asterisk* indicates *p* < 0.05 using a two-sided Students *t-*test. All images were taken at the same magnification. The *scale bar* is 20 microns

In LS8817 cells the number of ATRX foci began to subtly increase as soon as two days after addition of CDK4i. The number of foci continued to increase, reaching its maximum by day 7 (Fig. 2a). At seven days of drug treatment, ~70% of the ATRX foci overlapped with HP1γ foci, which are representative of the SAHF (Fig. 2b).

This was not cell line specific. Similar increases in the number of ATRX foci were also seen in LS0082 and LS141 cells that undergo CDK4i-induced senescence, but not in LS8313, LS7785-1, and LS8107 cells that fail to senesce in response to CDK4i but are quiescent (Fig. 2c).

This was not CDK4i specific. The number of ATRX foci also increased in senescent LS8817 cells following MDM2 knockdown (Supplementary Fig. 4A) or after treatment with doxorubicin (Supplementary Fig. 4B).

Importantly, ATRX foci and other indicators of senescence do not increase in all non-proliferating LS8817 cells. Neither an increase in ATRX foci nor an increase in SA-β-gal was observed in serum-starved LS8817 cells that exited the cell as determined by reduced BrdU incorporation (Supplementary Fig. 4C).

The accumulation of ATRX foci in senescent cells was not sarcoma specific. The number of foci increased in the SNB19 glioma cell line that undergoes CDK4i-induced senescence (Supplementary Fig. 5A). An increase in ATRX foci also is seen in A549 and H1975 non-small-cell lung cancer cell lines that undergo CDK4i-induced senescence but not in H358 in which CDK4i induces quiescence (Supplementary Fig. 5B). Additionally, while there was an increase in ATRX foci in CDK4i-treated MCF7 cells undergoing senescence, there was no increase in the number of foci when MCF7 cells quiesced after serum starvation (Supplementary Fig. 5C).

Thus, the increase in the number of ATRX foci was observed in broad range of cell types that underwent senescence.

**Fig. 3:**
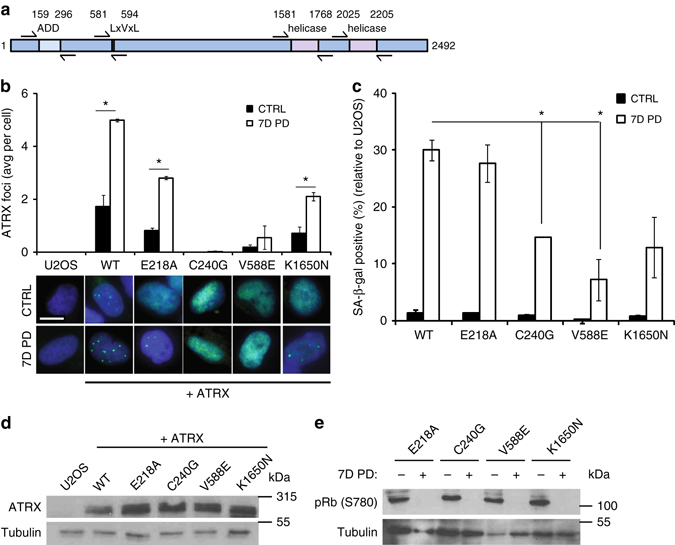
The ability of ATRX to support CDK4i-induced senescence in U2OS cells requires HP1 and H3K9me3 binding sites. **a** A schematic of ATRX indicating the domains and amino acid residues numbered as annotated on UniProt is shown. *Arrows* indicate the paired sequencing primers used to confirm the mutations. **b**–**d** U2OS cells were transfected with vectors expressing wild type or mutant ATRX alleles and stable transformants were selected with G418 and sorted to recover a GFP-low population as described in the methods. WT indicates wild type. **b** ATRX immunofluorescence was carried out in the mutants described in the legend to Fig. 2. The average number of ATRX foci per cell is plotted and representative images are shown. **c** The accumulation of SA-β-gal-positive cells was scored 7 days after PD0332991 (PD) treatment. *Asterisk* indicates *p* < 0.05 for the induction of SA-β-gal following treatment compared to cells expressing wild type ATRX. *p*-values were calculated using a two-sided Student’s *t-*test. **d** ATRX was detected with the A301-045A antibody. Tubulin is a loading control. **e** Rb phosphorylation, a measure of cell proliferation, was measured in the U2OS cells expressing different mutant ATRX constructs before and after treatment with PD0332991 (PD) for 7 days. Tubulin is a loading control. All data are represented as mean ± SEM from at least two independent experiments. See also Supplementary Fig. 10 for uncropped blots. All images were taken at the same magnification. The *scale bar* is 20 microns

To gain mechanistic insight into how ATRX foci formed during senescence, we addressed what domains of ATRX were required. Foci were readily detected following CDK4 inhibition in U2OS cells expressing the wild type ATRX allele; however, foci formation was reduced in cells expressing missense alleles in the H3K9me3 (C240G) and HP1 (V588E)-binding sites^42^ (Fig. 3b). Mutation of the H3K4me0-binding site, E218A, did not affect foci formation after CDK4 inhibition. The ability of the mutants to form foci after CDK4 inhibition was correlated with their ability to promote senescence, as measured by accumulation of SA-β-gal (Fig. 3c).

Additionally, the K1650N mutant, a hypomorphic mutation affecting helicase activity^47^, could be recruited into foci following drug treatment, but was unable to support the accumulation of SA-β-gal to levels seen in the cells expressing wild type ATRX (Fig. 3b and c). We also tried to assess the effect of eliminating ATRX/DAXX interaction but were unsuccessful. Expression of all of the mutant proteins was similar (Fig. 3d), and did not affect the ability of CDK4i to induce cell cycle exit, as measured by Rb phosphorylation (Fig. 3e). Thus, it is clear that both foci formation, driven through the H3K9me3- and HP1-binding sites, and the helicase activity were necessary for senescence.

### ATRX plays a role in the maintenance of the SAHF

ATRX can interact with PML^41^ and colocalized with PML in senescent LS8817 cells (Fig. 2b). Furthermore, the requirement for H3K9me3 and HP1 binding sites for ATRX to promote senescence and the colocalization of ATRX with HP1γ indicated that ATRX directly associated with the SAHF. Thus, we wanted to determine if these cytologically defined structures were affected by the loss of ATRX.

**Fig. 4:**
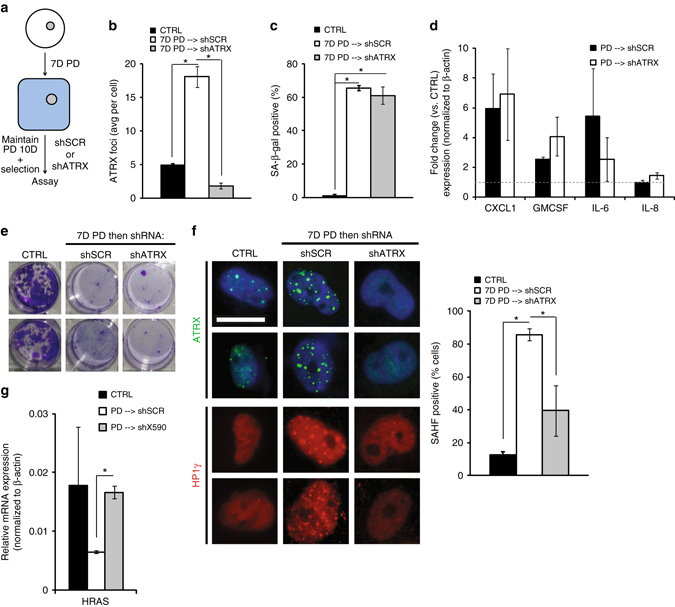
ATRX is required for maintenance of HP1γ SAHF in senescent cells. **a** LS8817 cells were treated as described. **b**–**d** Cells were then fixed and the number of ATRX foci (**b**), the accumulation of SA-β-gal-positive cells (**c**), and the accumulation of four mRNAs of the SASP cytokine program (**d**) were measured. **e** Clonogenic growth was assessed after cells were replated at low density the absence of drug. Colony number is not quantified as individual colonies could not be discerned. **f** The accumulation of HP1γ-positive cells (SAHF) was determined. Representative images are shown (*left*) and the percentage of SAHF-positive cells was quantified (*right*). **g** *HRAS* mRNA levels were analyzed by qPCR. All data are represented as mean ± SEM from at least three independent experiments. *Asterisk* indicates *p* < 0.05 using a two-sided Students *t-*test. All images were taken at the same magnification. The *scale bar* is 20 microns

In LS8817 cells, similar to what had been reported by others^22, 48^, PML-NBs began to accumulate before ATRX foci, within one day of drug treatment (Supplementary Fig. 6A). PML-NBs formed at similar levels in LS8817^shATRX^ and LS8817^scr^ cells treated with CDK4i (Supplementary Fig. 6B). PML-NBs remained qualitatively and quantitatively similar for the duration of our experiments. SAHF-positive cells first began to appear around 4 days after CDK4i were added and reached their maximum by day six. To determine if ATRX was required for the maintenance of PML-NBs and/or SAHF, we transduced senescent LS8817 cells that had been treated with CDK4i for seven days with a lentivirus expressing the ATRX hairpin (Fig. 4a). After selection for an additional 10 days in the presence of CDK4i, we measured accumulation of the PML-NBs, the accumulation of SA-β-gal and SAHF, the induction of the SASP mRNAs, and the irreversibility of arrest after removal of CDK4i. Loss of ATRX in a senescent cell did not reduce the number of PML-NBs (Supplementary Fig. 6B), the accumulation of SA-β-gal-positive cells (Fig. 4c), the expression of the SASP genes (some of which were even modestly enhanced, Fig. 4d)), or the irreversibility of growth arrest after CDK4i were removed as measured by long term clonogenicity after replating in the absence of the drug (Fig. 4e). However, the number of SAHF decreased (Fig. 4f). This indicated that ATRX was required for the maintenance of the SAHF in senescent cells.

### ATRX affects EZH2 and E2F targets during TIS

Previous efforts to describe the differences between genome wide expression in quiescent and senescent cells have used a single cell type treated with different inducers leading to the different outcomes^49, 50–53^. We thought that paired isogenic cell lines that respond differently to a single inducer might provide additional insight into the transcriptional networks that distinguish these cellular outcomes. Thus, we carried out genome wide RNA sequencing (RNA-seq) to identify genes differentially expressed in ATRX-deficient and wild-type LS8817 cells before and after the addition of CDK4i and sought to identify how ATRX affects the transition between quiescence and senescence.

**Fig. 5:**
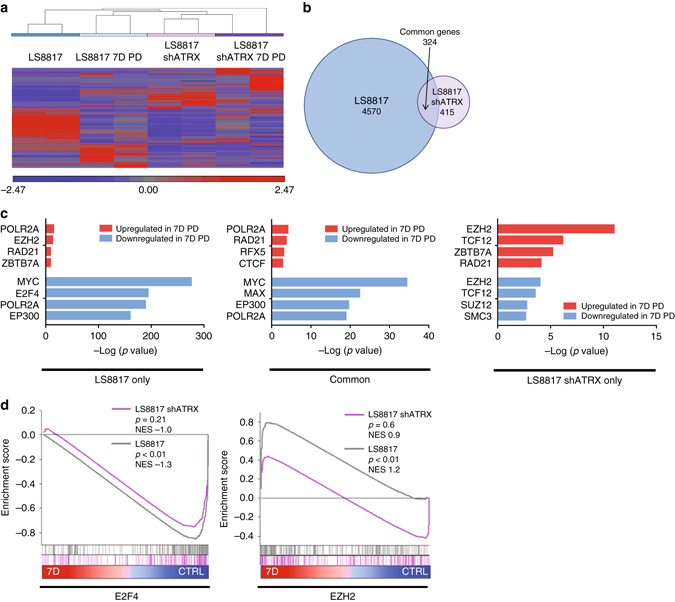
ATRX affects E2F and EZH2 target gene expression in senescent cells. **a** Hierarchical clustering based on expression of all RNAs sequenced by RNA-seq. Each row represents a single sample, and the conditions are indicated above; RNA-seq was performed in duplicate on each condition. **b** Venn Diagram indicating the number of genes that were differentially expressed with a fold change of at least −1.8 or 1.8 and an FDR < 0.05 across PD-treated vs. control samples in unperturbed and ATRX-deficient LS8817 cells; the genes that were found in common across both cell types are indicated. **c** Enrichr analysis of the top predicted transcription factors that regulate the upregulated and downregulated gene lists from **b**. The negative log of the *p*-value for the enrichment scores is plotted. **d** Gene set enrichment analysis (GSEA) was performed on across PD-treated vs. control samples in unperturbed and ATRX-deficient LS8817 cells, specifically analyzing E2F4 and EZH2 gene signatures. The profiles are shown with their corresponding normalized enrichment score (NES) and FWER *p*-values

It was not surprising that the changes in gene expression associated with CDK4 inhibition were dramatically affected by ATRX loss (Fig. 5a), as LS8817^shATRX^ cells underwent quiescence and LS8817 cells underwent senescence. Using analysis of variance (ANOVA) to interrogate which genes were up- or downregulated by at least a factor of 1.8 in untreated vs. treated cells and an FDR < 0.05 we found that there were 4894 genes whose expression changed significantly in LS8817 cells and only 739 in LS8817^shATRX^ knockdown cells (Fig. 5b). 324 of these genes overlapped. This indicated that ATRX plays an integral role shaping the gene expression program between quiescent and senescent cells induced by CDK4 inhibition.

We also used the Enrichr software^54^ to identify which transcription factor (TF) targets were specifically affected by the loss of ATRX (Fig. 5c). E2F4 target genes were enriched exclusively in the LS8817 cells, consistent with the hypothesis that ATRX is required to properly regulate E2F-dependent gene expression in SAHF. Myc, Max, and p300 target genes were enriched in both, as expected since both quiescent and senescent cells have exited the cell cycle. In the LS8817^shATRX^ cells, targets of SUZ12 and SMC3 were specifically downregulated following drug treatment.

EZH2 targets were upregulated following drug treatment in the LS8817 cells, whereas some EZH2 targets were upregulated and others were downregulated in LS8817^shATRX^ cells after CDK4 inhibition. This suggests a general perturbation of EZH2 target gene expression as a result of ATRX loss.

We used gene set enrichment analysis (GSEA) to compare the complete transcriptomes of drug treated cells to their asynchronous counterparts in both ATRX proficient and deficient populations. Of the aforementioned gene sets, only E2F4 genes were significantly enriched in the cycling population, while only EZH2 genes were significantly enriched in the senescent population (Fig. 5d). In the LS8817^shATRX^ drug treated quiescent cells, neither E2F4 nor EZH2 gene sets were not significantly enriched compared to cycling cells. Thus, ATRX is important for their appropriate regulation during CDK4i-induced senescence.

### *HRAS* is a direct target of ATRX repression during TIS

Overall changes in gene expression could reflect both direct and indirect contributions of ATRX. Thus, we wanted to identify a gene (or genes) directly bound and regulated by ATRX in senescent cells that might be important for senescence. In order to gain this insight and simultaneously identify common direct roles of ATRX shared in doxorubicin and CDK4i-induced senescent cells we performed chromatin immunoprecipitation followed by sequencing (ChIP-seq) in LS8817 cells 7 days after drug treatment. To identify binding sites specifically related to senescence, we also carried out ChIP-seq in quiescent serum starved and cycling LS8817 cells. Confirming the specificity of the immunoprecipitation, antibodies to ATRX failed to pull down sufficient DNA from U2OS cells with which to prepare a library.

**Fig. 6:**
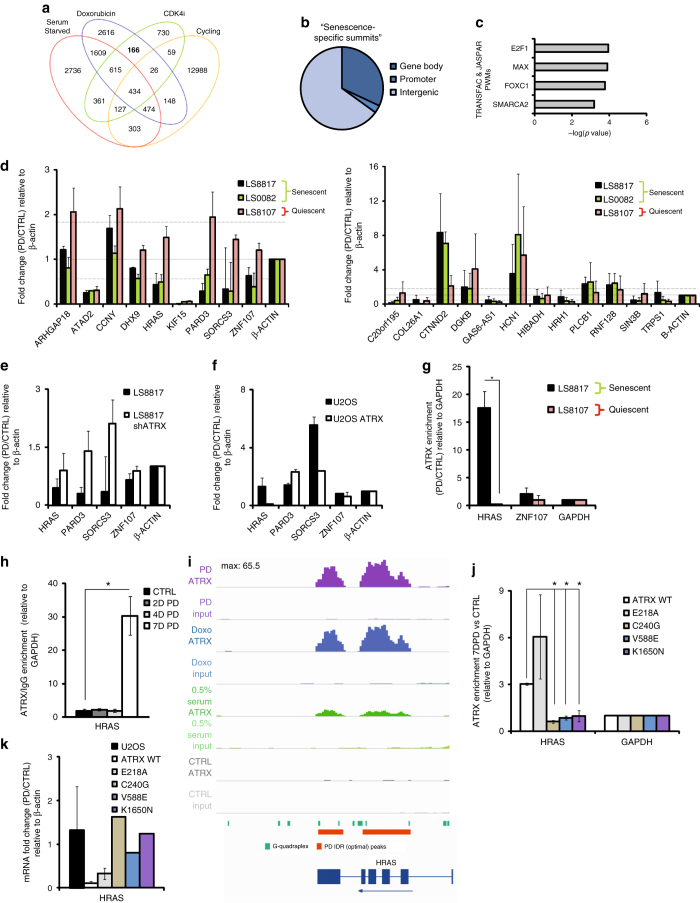
ATRX directly binds to and represses *HRAS* in response to CDK4 inhibition. **a** Venn diagram indicating the number of ATRX-specific summits identified by ChIP-sequencing performed in untreated (cycling) LS8817 cells, senescent LS8817 cells treated with either PD0332991 for 7 days (CDK4i) or doxorubicin for 7 days, and quiescent cells induced by growth in low serum for 5 days (0.5% serum starved). **b** Pie chart summarizing the distribution of the 166 senescence-specific summits within gene bodies, associated with promoters or in intergenic regions. **c** Enrichr analysis of the top predicted transcription factors that regulate the “gene body” and “promoter” associated genes from **b**. **d** Expression of the indicated genes downregulated in senescence (*left*), or upregulated in senescence (*right*) in the cell lines treated with CDK4i was measured by qPCR and plotted as a fold change in treated (7 days) cells compared to untreated cells, normalized to β-actin. *Dashed lines* are drawn at 1.8 fold changes. **e**, **f** The expression of the indicated genes was measured in the indicated cell lines in which ATRX levels were manipulated (**e**, LS8817; **f**, U2OS). **g** ATRX ChIP experiments were performed in untreated controls and PD0332991-treated cells and the relative binding of ATRX at the indicated loci was analyzed by qPCR. **h** ATRX ChIP was performed in LS8817 cells treated with PD0332991 at the indicated times and enrichment at the *HRAS* locus was determined by qPCR is plotted. **i** Genome browser view of ATRX enrichment at the *HRAS* locus under different growth conditions. The *red bars* below the tracks indicate the peaks called by the IDR algorithm. *Green bars* indicate sequences predicted to form G-quadruplex structures. *Blue boxes* represent the exonic structure of *HRAS*. Graphs are obtained from one of the two biologic ChIP-seq replicates but are representative of both. **j** ATRX was immunoprecipitated from CDK4 inhibitor treated and untreated U2OS cells in which either wild type (WT) or mutant proteins were expressed and enrichment at the *HRAS* locus determined by qPCR. **k** *HRAS* mRNA levels were measured in the cells described in Fig. 3. All data are represented as mean ± SEM from at least two independent experiments. *Asterisk* indicates *p* < 0.05 using a two-sided Student’s *t-*test

ATRX-binding sites were identified using the irreproducible discovery format framework. Reported telomeric, centromeric and repetitive regions were identified in all samples and were filtered out of subsequent analyses. While there were many inducer-specific binding sites, we focused on the 166 sites shared by both doxorubicin and CDK4i and not found in serum starved or cycling cells (Fig. 6a). One-third of these were in gene bodies or promoters (Fig. 6b) representing 41 unique genes (Table 1). The remaining sites were in intergenic sequences (Supplementary Table 1). This ratio is consistent with that previously reported by others^33, 55^. We were unable to discern any relationship between the distribution of the intergenic sites along the chromosomes relative to centromeres, telomeres, or other binding sites. Likewise, we were also unable to discern a common structural or sequence element in these intergenic regions, including G-quadruplexes that are enriched at ATRX binding sites^55^. Intergenic sequences were also not related to long non-coding RNAs, which might be involved in chromosome wide repression.

**Table 1:** RNA expression of genes bound by ATRX in senescent cells

	

RNA expression repressed in senescent cells is indicated in green

RNA expression induced in senescent cells is indicated in brown

Shading indicates the degree of repression or activation.

Fold repression/induction are indicated by the numbers

n.d., not determined

Ratios of gene expression in CDK4i-induced or doxorubicin-induced senescent cells compared to either cycling untreated cells or quiescent cells induced by serum starvation are shown. Whether each gene belongs to the TRANSFAC/JASPAR PWM transcription factor target gene sets as described in Fig. 6c is indicated

Consistent with the regulation of SAHF by ATRX we noted that there was a predominance of E2F binding sites in this subset of 41 genes (Fig. 6c and Table 1). Additionally, this subset was also enriched for MAX and FOXC1 binding sites.

39 of these 41 core genes were identified in the RNA-seq data (GEO accession number GSE74620). 18 of the 39 were similarly regulated when comparing expression in both the CDK4i and doxorubicin treated cells with expression in cycling or serum starved cells (Table 1). Eight were decreased in at least 3 of the comparisons (*KIF15*, *ATAD2*, *DHX9*, *ARHGAP18*, *PARD3*, *HRAS*, *ZNF107*, and *SORCS3*), and 10 were increased in all 4 of the comparisons (*RNF128*, *PLCB1*, *HCN1*, *CTNDD2*, *TRPS1*, *COL26A1*, *GAS6-AS1*, *SIN3B*, *HIBADH*, and *C20orf195*).

Only four of these were observed in another senescent cell, but not in a quiescent cell. *HRAS*, *PARD3*, *ZNF107*, and *SORCS3* were also repressed in CDK4i-treated senescent LS0082 cells and not in CDK4i-treated quiescent LS8107 cells (Fig. 6d). *HRAS*, *ZNF107*, and *SORCS3* were also de-repressed following treatment of isogenic LS8817 cells in which ATRX was reduced (Fig. 6e), and repressed following treatment of isogenic U2OS cells expressing ATRX (Fig. 6f). *PARD3* was not repressed in U2OS^ATRX^ cells treated with CDK4i. Changes in *ZNF107* were consistent with our expectations but more modest than changes in *HRAS* or *SORCS3*.

We next confirmed that ATRX was bound to the *HRAS* and *ZNF107* loci using ChIP-qPCR (Fig. 6g). ATRX binding was strongly enriched at the *HRAS* locus in senescent LS8817 but not quiescent LS8107 cells. Binding was more modestly enriched at the *ZNF107* locus. We could not design primers to evaluate binding to the *SORCS3* locus because the sequences identified by ChIP-seq were AT-rich.

To determine if the repression of *HRAS* and *ZNF107* were sarcoma specific, we looked at their expression in senescent SNB19 glioma cells, senescent A549 and H1975 lung cancer cells and quiescent H358 lung cancer cell lines (Supplementary Fig. 7). *HRAS* expression was consistently reduced in the three senescent cells and not the quiescent cell, whereas *ZNF107* was not. Thus, repression of *HRAS* following CDK4 inhibition was not specific to sarcoma cell lines.

ATRX binding to the *HRAS* locus was first detected seven days following drug treatment (Fig. 6h). This locus contains a number of G-quadraplex forming sequence elements and ATRX was bound over the entire locus but enriched over the exonic sequences (Fig. 6i). ATRX binding was clearly enriched in senescent cells as compared to serum starved cells (Fig. 6i). Consistent with the importance of ATRX for repression, *HRAS* expression increased when ATRX was knocked down in cells that were already senescent (Fig. 4g) and was not reduced in LS8817^shATRX^ shX588 cells treated with CDK4i or doxorubicin (Supplementary Fig. 1F). In the U2OS cells expressing the different alleles of ATRX, *HRAS* binding was compromised by C240G, V558E, and K1650N mutations but not the E218A mutation (Fig. 6j). Consistent with this, *HRAS* mRNA expression was repressed following CDK4 inhibition in cells expressing wild type or the E218A mutant but not those expressing the C240G, V588E, or K1650N mutants (Fig. 6k).

Given that the increase in ATRX foci first occurs within two days after CDK4i was added to these cells, and *HRAS* was bound and repressed at later times, once the number of foci increased to its maximum, we suggest that the gene expression program(s) regulated by ATRX during senescence were dynamically evolving.

### Manipulating *HRAS* expression affects geroconversion

**Fig. 7:**
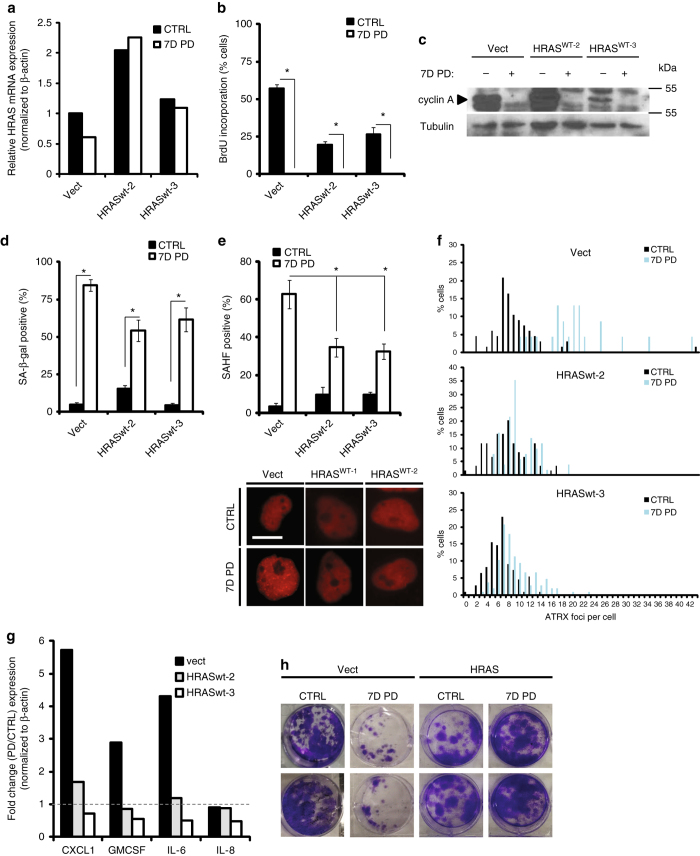
Stabilizing *HRAS* expression prevents the transition from quiescence to senescence. **a**–**h** Wild-type *HRAS* (HRASwt) or a vector control (vect) was stably expressed in LS8817. After 4 days of selection with puromycin, cells were treated with 1 μM of PD0332991 for seven additional days (7D PD) and compared to cycling (CTRL) cells and analyzed for the following: *HRAS* mRNA levels measured by qPCR (**a**), growth arrest by BrdU incorporation (**b**) and cyclin A immunoblot (**c**), SA-β-gal (**d**), HP1γ SAHF (**e**), number of ATRX foci on an individual basis (**f**), induction of four SASP factors as measured by qPCR (**g**), and ability to return to cell cycle once PD0332991 once the dug was removed (**h**). Representative SAHF images are shown below panel e. Colony formation in panel h was not quantified as individual colonies could not be distinguished. Individual experiments are presented and all data are represented as mean ± SEM from three independent fields of view. *Asrterisk* indicates *p* < 0.05 using a two-sided Student’s *t-*test. All images were taken at the same magnification. The *scale bar* is 20 microns. See also Supplementary Fig. 10 for uncropped blots

We next measured the effect of blocking CDK4i-induced downregulation of *HRAS* in LS8817 cells. After exhaustive screening we were able to obtain two independent replicates in which the enforced expression of *HRAS* was both near physiologic level and not repressed after the cells were treated with CDK4i for seven days (Fig. 7a). These cells proliferated more slowly than control cells expressing a mock vector (Fig. 7b). Seven days after PD0332991 was added the cells exited the cell cycle (Fig. 7b and c), and although the accumulation of SA-β-gal-positive cells was lower in the *HRAS* expressing cells it was not significantly different than in the control cells (Fig. 7d). On the other hand, both HP1γ positive cells (Fig. 7e) and the number of ATRX foci per cell (Fig. 7f) were reduced compared to the control cells. However, the number of ATRX foci was increased modestly, similar to the extent seen in parental LS8817 cells treated with CDK4i for just two days (Fig. 2a). The expression of CXCL1, GM-CSF, and IL-6 mRNAs, three markers of the CDK4i-induced SASP, were not increased in the HRAS expressing cells (Fig. 7g). *HRAS* expressing cells were also more capable of returning to growth after removal of CDK4i (Fig. 7h). Consequently, enforced expression of *HRAS* can prevent CDK4i TIS, indicating that down-regulation of *HRAS* plays a role establishing the terminal senescent state, particularly with regard to the accumulation of both ATRX foci and HP1γ SAHF.

**Fig. 8:**
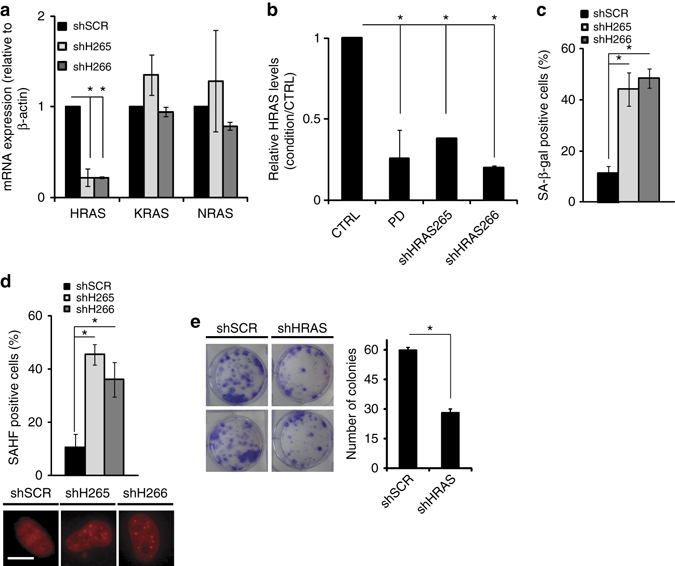
Reducing *HRAS* expression can drive quiescent cells into senescence. **a** *HRAS* was stably knocked down with two independent hairpins each in cycling LS8817 cells and transcript levels were measured by qPCR. Expression levels of the indicated genes were individually normalized to 1 in the shSCR cells. **b**–**e** LS8817 cells were serum starved for 3 days and subsequently infected with the indicated shRNA encoding lentiviruses. After an additional 5 days of selection, the accumulation of *HRAS* mRNA (**b**), SA-β-gal (**c**), and SAHF (**d**) were measured. Representative SAHF images are shown (**e**). Long-term clonogenicity was measured by crystal violet staining of colonies 3 weeks after the cells were replated in complete medium. Representative images are shown (*left*) and colony numbers are quantified (*right*). all data are represented as mean ± SEM from three independent experiments. *Asterisk* indicates *p* < 0.05 using a two-sided Students *t-*test. All images were taken at the same magnification. The *scale bar* is 20 microns

We also asked whether suppressing *HRAS* in a quiescent cell would induce progression into senescence. To determine this, we first identified two independent shRNAs that specifically repress *HRAS* expression and did not grossly affect expression of *KRAS* or *NRAS* expression in LS8817 cells (Fig. 8a). The extent of *HRAS* repression was similar to that seen following treatment with CDK4i (Fig. 8b). We then serum starved LS8817 cells into quiescence for 2 days, after which we maintained serum starvation and knocked down *HRAS*. Seven days later we measured the appearance of SA-β-gal-positive cells, HP1γ foci-positive cells and the ability of the cells to return to the cell cycle when serum was restored. In cells transduced with the shRNA targeting *HRAS*, but not in those expressing a scrambled control shRNA, the number of SA-β-gal-positive and HP1γ-positive cells increased (Fig. 8c and d). Growth was reduced following restoration of serum compared to control cells (Fig. 8e). Because serum starvation induced the accumulation of the mRNAs of the SASP we were not able to assess their expression as a marker of senescence.

We also treated LS8107 cells with CDK4i to induce quiescence and then reduced *HRAS* (Supplementary Fig. 8). Reducing *HRAS* expression in these cells (Supplementary Fig. 8A) increased the number of SA-β-gal-positive (Supplementary Fig. 8B) and HP1γ foci-positive cells (Supplementary Fig. 8C). The number of ATRX foci also increased (Supplementary Fig. 8D). As expected because of the cell type specific nature of the SASP, the expression of the SASP mRNAs was different than we had seen in LS8817 cells. LS8107 cells had very little GM-CSF expression under any condition (Supplementary Fig. 8E). However, the expression of CXCL1 and IL-8 mRNA was induced in those cells in which *HRAS* was knocked down compared to the level of expression in those cells that remained quiescent after using a scrambled vector. IL-6 mRNA was elevated in all conditions compared to cycling control cells. Thus, even given the cell type specificity associated with the SASP, there is evidence that knocking down *HRAS* can induce elaboration of this program of gene expression as well. Collectively these data indicate that the loss of *HRAS* can combine with CDK4 inhibition to drive quiescent cells into a state of senescence.

## Discussion

Senescence is a preferred outcome of cytostatic chemotherapy because these cells are stably withdrawn from the cell cycle and may elaborate a secretory program that can recruit cells of the immune system, resulting in tumor clearance^56, 57^. The development of small molecule CDK4i has made it possible to learn about the molecular events that occur as reversible quiescence matures into a stable state of senescence. Here we report that the chromatin remodeling enzyme ATRX is required for quiescent cancer cells to progress into senescence triggered by MDM2 loss and by the treatment of cells with CDK4i or doxorubicin, a p53-independent and a p53-dependent DNA-damage associated pathway, respectively. ATRX plays a role in the maintenance of the SAHF and the regulation of *HRAS* gene expression. These events depend on the ability of ATRX to bind to H3K9me3 histones and HP1. We discuss the significance of the role of ATRX in SAHF and the implications of repressing *HRAS* for the design of combination chemotherapies with CDK4i.

The mechanism of SAHF formation has been extensively reviewed^18–20^. Although SAHF are not observed in all contexts of cellular senescence, when they do form they are required for senescence. Loss of assembly factors and structural components prevent cells from exiting the cell cycle and undergoing senescence when stressed. For example, loss of Asf1, HMGA, and Rb can prevent the formation of SAHF in response to oncogenic RAS in normal human diploid fibroblasts, and cells lacking any one of these genes continue to proliferate when challenged with RAS^21–23^. Here we have shown that ATRX is required for the formation and maintenance of the SAHF. ATRX interacts and co-localizes with PML-NBs (refs ^40, 41^ and Fig. 4a), and its ability to interact with HP1 and H3K9me3 histone, are important for senescent cells to maintain SAHF and repress HRAS.

However, unlike the other proteins that regulate SAHF, the absence of ATRX, does not cause continued cell proliferation. Thus ATRX is a unique regulator of the SAHF. Given the requirements for both HP1 and H3K9me3 binding sites, it is not only likely that ATRX is required after H3.3 is deposited and modified into a K9me3 state but these interactions may be individually responsible for association and the stabilization of ATRX at the SAHF. Regardless of the precise mechanistic details that remain to be determined, it is clear that the SAHF are dynamic and actively maintained in senescent cells.

While SAHF are required for entry into senescence, they do not seem to be required for its maintenance. Eliminating SAHF in a senescent cell did not affect the other hallmarks of senescence. We suggest that redundant mechanisms maintain the senescent state. One such mechanism might be related to the SASP and downstream signaling programs. It will be interesting to determine if other regulators, important for becoming senescent, are also less important for its maintenance.

In addition to an indirect role for ATRX in MDM2 turnover early after treatment with CDK4i^35^, a direct role for ATRX during CDK4i TIS is supported by its binding to *HRAS*, well after MDM2 levels go down and concomitant with its role maintaining SAHF. An increase in ATRX foci is first detected soon after cells exit the cell cycle and correlates with the timing of MDM2 turnover; however, the identity of the ATRX binding sites must evolve over time as cells progress into senescence. Identifying these programs may provide additional insight into this transition.

ATRX represses expression from the chromosomal *HRAS* locus during TIS. Reducing *HRAS* is sufficient to drive a quiescent cell into senescence as marked by the accumulation of SA-β-gal, HP1γ SAHF and stable growth arrest, and enforced expression of *HRAS* could prevent progression of cells into a stable senescent state and prevent the increase in ATRX foci formation. This is consistent with other findings that reported that repression of RAS signaling is required to establish the senescent state in cell lines and mouse models^58–62^. Consequently, the loss of ATRX during the development of tumors and its repression of *HRAS* expression may contribute to overcoming senescence, similar to how more well known mutations in the feedback loops affect signaling flux through the RAS pathway^63, 64^.

The importance of reducing *HRAS* to achieve senescence might have clinical implications. MEK inhibitors have been shown to have a broader benefit when combined with CDK4 inhibition in a variety of circumstances (reviewed in ref. ^65^). These drugs can modulate signaling flux through the RAS pathway, which is critical for accumulation of D-type cyclins and formation of cyclin D-CDK4 complexes. One way to explain this synergy is to suggest that such drugs dampen the increase in cyclin D levels induced by CDK4 inhibition, thus enforcing cell cycle exit (reviewed in ref. ^66^). Although not mutually exclusive, an equally compelling possibility is that these inhibitors might drive CDK4i-induced quiescent cells into senescence. Senescence would not only improve the durability of cell cycle exit, which is particularly important given that patients are intermittently withdrawn from the CDK4i drugs Palbociclib and Ribociclib to preserve hematopoietic function, but it may also enhance the clearance of the tumor by mobilizing the immune response through the elaboration of the SASP^56^. Thus, sequencing CDK4 and Ras inhibition might offer a new paradigm for combining these drugs for therapeutic purposes.

## Methods

### Cell culture

All of the WD/DDLS cell lines used in this manuscript were generated from patient-derived surgical samples from Samuel Singer’s lab. All the cell lines bear amplification of the *CDK4* and *MDM2* oncogenes, are wild type for Rb and wild type p53, and their capacity to undergo apoptosis or cell cycle exit in response to Nutlin-3a has been confirmed^35, 67, 68^.

All other cell lines were obtained from the ATCC. All cell lines were maintained in DME HG supplemented with 10% heat-inactivated fetal bovine serum (unless otherwise indicated) and 2 mM l-glutamine.

### Senescence analyses

SA-β-gal was assayed using the Cell Signaling Technologies kit (#9860) according to manufacturer’s instructions. SAHF was assayed via HP1γ immunofluorescence with antibodies as listed above. We only consider cells with ≥ 5 HP1γ foci as SAHF-positive. Clonogenic growth arrest was assayed by treating cells as indicated in the corresponding figure legend(s) and then trypsinizing and re-plating cells in complete, drug-free media. Cells were allowed to grow for 21 days prior to staining with crystal violet. Statistical comparisons of these senescence markers were performed using a two-sided Students *t-*test.

The SASP is inducer and cell type specific cytokine secretion program^13^. We identified markers of the LS8817 SASP program by probing R&D Systems Human Cytokine Arrays with tissue culture medium from untreated control and seven day CDK4i-treated LS8817 cells there was a two-fold or greater increase in the amount of secreted CXCL1, GM-CSF, IL-6 and IL-8 cytokines (Supplementary Fig. 9) after drug treatment. Seven days after drug addition had previously been shown to be sufficient for the cells to acquire other senescent hallmarks^35^. These cytokines were not increased in LS8817^shATRX^ cells treated with PD0332991 that undergo quiescence^35^. Thus, these cytokines are representative of the SASP induced in these cells by the CDK4i PD0332991.

Although it is known that mRNA expression does not always reflect the secretion of the cytokines (Lujambio, personal communication), we developed a four cytokine mRNA panel to assess the SASP in the LS8817 cells and their derivatives in which gene expression was altered. There was an approximately two-fold or greater increase in the expression of CXCL1, GM-CSF and IL-6 mRNA but no significant change in the expression of IL-8 mRNA.

### Antibodies

Antibodies were as follows (catalog number and immunoblot diltuions (if applicable) in parentheses): MDM2 (SMP14, 1:500), total Rb (IF8, 1:200), cyclin A (H432, 1:1000), p16 (C20, 1:1000), p53 (DO-1 and Bp53-12, 1:500), tubulin (C20, 1:2000), ATRX (H-300, 1:1000 and D-5, 1:500), PML (PG-M3), and normal rabbit IgG (SC-2027) were obtained from Santa Cruz Biotechnology, phospho-Rb 780 (#9307, 1:1000) from Cell Signaling, HP1γ (05-690) and γH2AX (05-636) from Millipore, ATRX (A301-045A, 1:2000) from Bethyl Laboratories, 53BP1 (ab172580) and LC3 (ab48394) from Abcam, GFP (A11122) from Life Technologies. Dilutions for immunofluorescence were as follows: ATRX (A301-045A) 1:2000; HP1γ (05-690) 1:5000; PML (PG-M3) 1:1000; γH2AX (05-636) 1:200; 53BP1 (ab172580) 1:2000; GFP (A11122) 1:1000. Uncropped versions of immunoblots are provided in Supplementary Fig. 10.

### Generation of and re-analysis of ATRX mutants

ATRX was mutated using standard QuikChange procedures and Platinum® *Taq* DNA Polymerase, High Fidelity (ThermoFisher) according to manufacturer’s protocols. PCR products were then digested with DpnI (New England Biolabs) according to manufacturer’s protocols before being transfected into ElectroMAX^TM^ Stbl4^TM^ Competent Cells (ThermoFisher) according to manufacturer’s protocols.

ATRX was delivered into U2OS cells using a full-length, N-terminally GFP-tagged construct generously provided by David Picketts. Briefly, U2OS cells were transfected using siLentFect Lipid Reagent (BioRad) according to manufacturer’s protocols. 48 h following transfection, cells were selected using G418 (500 µg/ml) for 5 days. Selected cells were sorted using fluorescent activated cell sorting on a MoFlow sorter (Beckman Coulter) and untransfected U2OS cells were used to mark the GFP-negative population. A GFP-low population was collected.

To verify the integrity of mutant DNA in transfected cells, we extracted genomic DNA (QIAGEN DNEasy) and amplified and sequenced specific regions of ATRX indicated in Fig. 2a (see Supplementary Table 2 for primers).

### Telomere restriction fragment length assay

Genomic DNA was isolated from the U2OS and U2OS^ATRX^ cells using DNeasy kit (QIAGEN) according to manufacturer’s protocols and telomere length determined using the *T*elo*TAGGG* telomere Length Assay (Roche) according to manufacturer’s protocols. Briefly, 1 μg of DNA was digested using with *Hinf*I and *Rsa*I, resolved on agarose gels, transferred onto a nylon membrane, and hybridized with a digoxigenin-labeled telomere-specific TTAGGG probe. Fragments were visualized via chemiluminescence.

### Gene targeting by shRNA

shRNA were delivered in the pLKO.1 vector (Open Biosystems) and infected cells selected using puromycin (1 μg/ml); infection with a virus carrying a scramble control (5′-CAACAAGATGAAGAGCACCAA-3′) was used as a control in all experiments utilizing shRNA. shRNA for *MDM2* (380, 5′-TACTAGAAGTTGATGGCTGAG-3′) and *ATRX* (588, 5′-GCCTGCTAAATTCTCCACATT-3′; 590, 5′-CGACAGAAACTAACCCTGTAA-3′ were previously characterized^35^. Lentiviral vectors targeting *HRAS* were made with the following sequences (265, 5′-CGGAAGCAGGTGGTCATTGAT-3′; 266, 5′-GTGTGTGTTTGCCATCAACAA-3′),

### RNA sequencing

RNA was extracted from cells treated as described using the manufacturer’s protocol (RNeasy, QIAGEN). All RNA-seq experiments were performed on three biologic replicates except for LS8817^shATRX590^ cells, which were performed in duplicate. RNA quality was checked on a BioAnalyzer to ensure a minimum RNA Integrity Value (RIN) of 7. Libraries were generated using 500 ng of input RNA per sample according to the manufacturer’s instructions for TruSeq mRNA Library Prep Kit V2 (Illumina) with 8 cycles of PCR. Libraries were pooled and run on an Illumina HiSeq 2500, high output, to obtain 30 million paired end, 50 nucleotide-long reads. The RNA-Seq reads were aligned to the human reference sequence hg19 with the RNASeq aligner STAR (version_2.4.0c). Genes annotated in Gencode version 18 were quantified with featureCounts (subhead package version 1.4.3-p1). The raw counts were then subjected to the Bioconductor package DESeq2 to call for differential expression between the groups of samples. Enrichment of differential expression in sets of genes was determined using Gene Set Enrichment Analysis (GSEA) on gene groupings from MSigDB, as well as custom sets (Subramanian et al, 2005). RNA-seq data was deposited on the Gene Expression Omnibus (GEO, http://www.ncbi.nlm.nih.gov/geo/) under the accession number GSE74620.

### RNA sequencing gene list derivation

RNA sequencing data analysis and comparisons were performed with Partek Software. The gene lists included all genes that showed at least a 1.8 fold change (FDR < 0.05) when comparing control and 7 day PD0332991-treated samples.

### Transcription factor profiling and GSEA

Gene lists were analyzed using the publicly available Enrichr software (http://amp.pharm.mssm.edu/Enrichr/)^53^ and the top four most significantly enriched groups (based on *p*-value) were reported. For GSEA, gene lists were derived from the RNA-seq data and were compared against gene lists in the publicly available Molecular Signatures Database (MSigDB) v5.1. The specific gene sets analyzed were KONDO_EZH2_TARGETS (M5301), V$E2F4DP1_01 (M10526). GSEA statistical analysis was carried out with publicly available software from the Broad Institute (http://www.broadinstitute.org/gsea/index.jsp)^69^.

### Real-time quantitative PCR

cDNA was synthesized from 1 μg of each RNA sample (extracted as above) using the One *Taq* RT-PCR Kit and olio-dT primers (New England BioLabs). cDNA was diluted 1:5 and 1 μl of reaction was used for qPCR using 400 nM of each forward and reverse primers and SYBR Green PCR Master Mix (Life Technologies) according to manufacturer’s protocols. qPCR was performed on Viia 7 Real-Time PCR System (Thermo Scientific). All primers are listed in Supplementary Table 2. All real-time quantitative PCR (RT–qPCR) comparisons are by two-sided *t*-test.

### Chromatin immunoprecipitation followed by sequencing

ATRX ChIP was performed as previously described^54^. Briefly, cells were fixed with 2mM EGS (Pierce 26103) for 45 min at room temperature in PBS, followed by 1% formaldehyde for an 20 additional minutes at room temperature. The reaction was quenched with 125 mM glycine and samples were sonicated to ~ 300 bp. Lysates were clarified by centrifugation and immunoprecipitated with ATRX H-300 antibody or rabbit IgG. Barcoded Illumina libraries were generated using the Kapa Hyper Prep Kit (Kapa Biosystems, Wilmington, Massachusetts) according to the manufacturer’s instructions, with 12 cycles of PCR amplification. Libraries were pooled and run on an Illumina HiSeq 2500, v4 chemistry, to obtain 30–40 million single read, 50 nucleotide-long reads passing filter. Reads were adapter and quality trimmed using Trim Galore! (http://www.bioinformatics.babraham.ac.uk/projects/trim_galore), aligned with BWA mem v0.7.8^70^, and realigned around indels and base quality score recalibrated with the Genome Analysis Toolkit v3.1.1^71^. This was repeated twice with biologic replicates at different times.

MACS v2.0.10^72^ was used to call peaks within the irreproducible discovery rate (IDR) framework (https://www.encodeproject.org/software/idr/; https://sites.google.com/site/anshulkundaje/projects/idr). Peaks were annotated with the most proximal upstream and downstream genes (hg19 RefSeq genes) filtered for technical false positives and intergenic sequences, and then overlapped with −5 to +1 kb promoters. Known telomeric, centromeric, and repetitive sequences were also annotated within the peaks. Genomic track images were generated using Integrative Genomics Viewer^73^. ChIP-seq data was deposited on the Gene Expression Omnibus (GEO, http://www.ncbi.nlm.nih.gov/geo/) under the accession number GSE74619.

### Data availability

All sequencing data from this study have been deposited in the National Center for Biotechnology Information Gene Expression Omnibus (GEO, http://www.ncbi.nlm.nih.gov/geo/) and are accessible through following accession numbers: RNA-seq GSE74620 and ChIP-seq GSE74619. All other data are available from the corresponding author on request.

## Electronic supplementary material

Supplementary Information
