# Supplementary material for: ATRX is a regulator of therapy induced senescence in human cells
Source: Nat Commun. 2017 Aug 30;8:386. doi: 10.1038/s41467-017-00540-5 (PMC5577318; doi:10.1038/s41467-017-00540-5)
Supplement: Supplementary file 1 — Supplementary Information [file 41467_2017_540_MOESM1_ESM.pdf]

Description of Supplementary Files

Title: Supplementary Information

Description: Supplementary Figures and Supplementary Tables

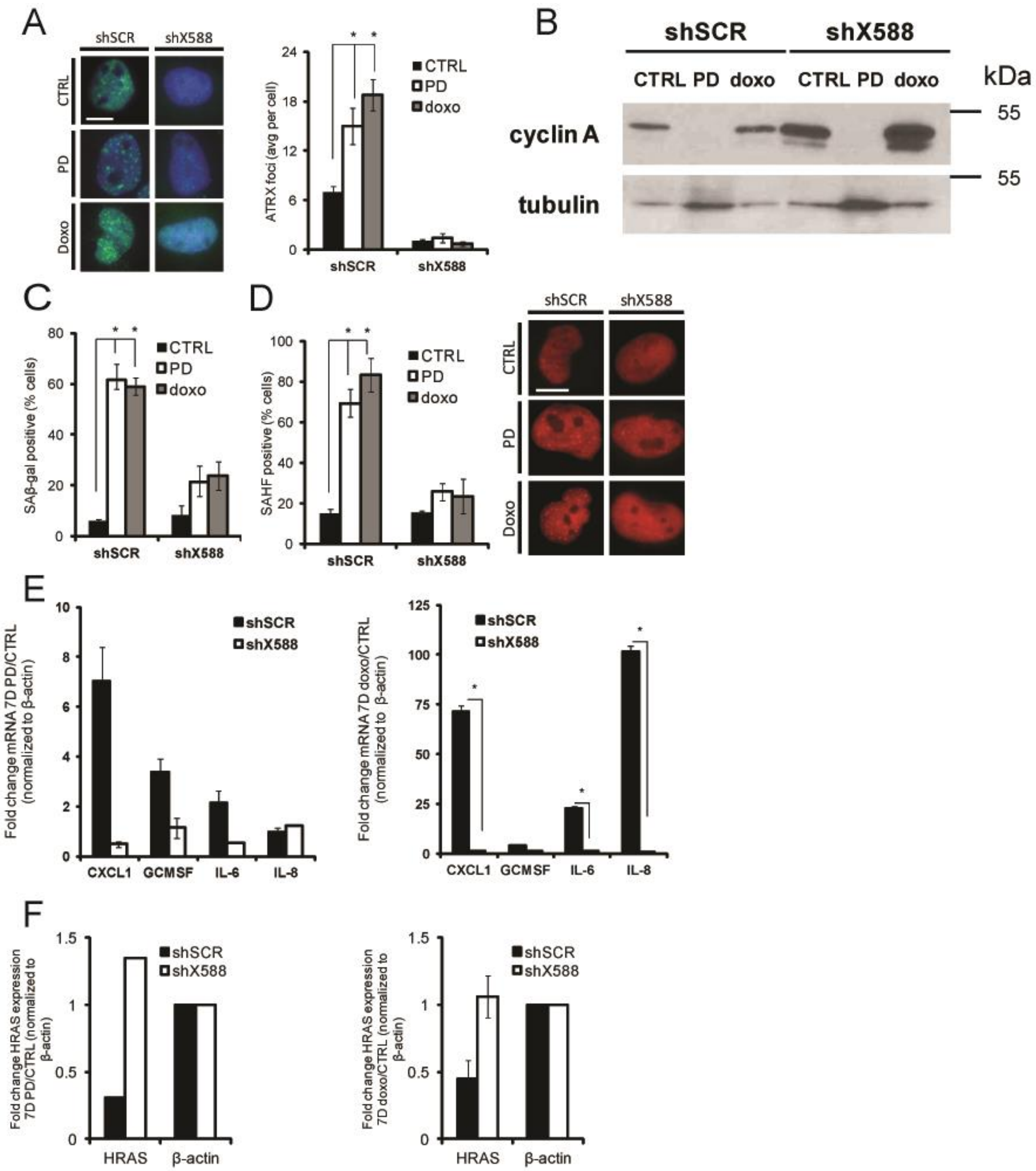

Supplementary Figure 1. Reducing ATRX suppresses CDK4i and doxorubicin induced senescence in LS8817 cells. LS8817^{scr} and LS8817^{shATRX} cells were generated as described in the legend to Figure 1, but this time using a different shRNA targeting ATRX (shX588). The cells were treated with either 1μM PD0332991 or 100nM doxorubicin for seven days. **(A)** Knockdown was confirmed by immunofluorescence. Representative images are shown (left) and foci were quantified (right). **(B-F)** The accumulation of cyclin A was measured by immunoblot (B), accumulation of SA-β-gal (C) and SAHF (D) by microscopy and immunofluorescence, and accumulation of SASP (E) and *HRAS* (F) mRNAs by qPCR. Representative SAHF images are shown in panel D. All data are represented as mean ± SEM from at least 3 independent experiments. * indicates $p < 0.05$ using a two-sided Students *t*-test. Scale bars indicate 20 microns.

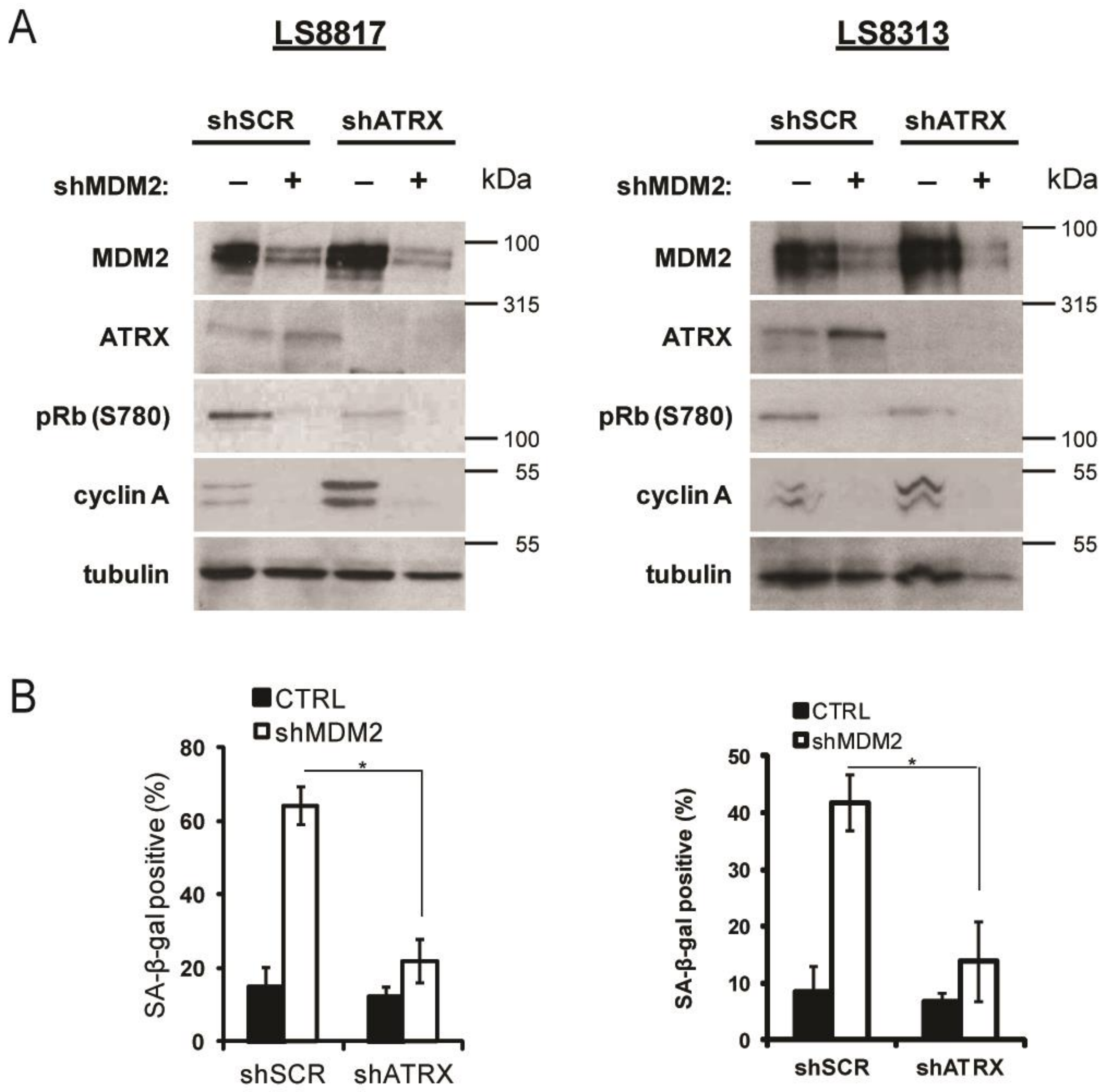

Supplementary Figure 2. ATRX is required for senescence induced by MDM2 knockdown. The LS8817^{scr} and LS8817^{shATRX} cells (left) were described in Figure 1. Similarly generated LS8313^{scr} and LS8313^{shATRX} cells (right) were prepared. **(A-B)** These cells were transduced with an shRNA vector targeting MDM2 and the effect on protein expression (A) and accumulation of SA-β-gal (B) were determined. ATRX was detected with the D-5 antibody. All data are represented as mean ± SEM from at least 3 independent experiments. * indicates $p < 0.05$ using a two-sided Students *t*-test.

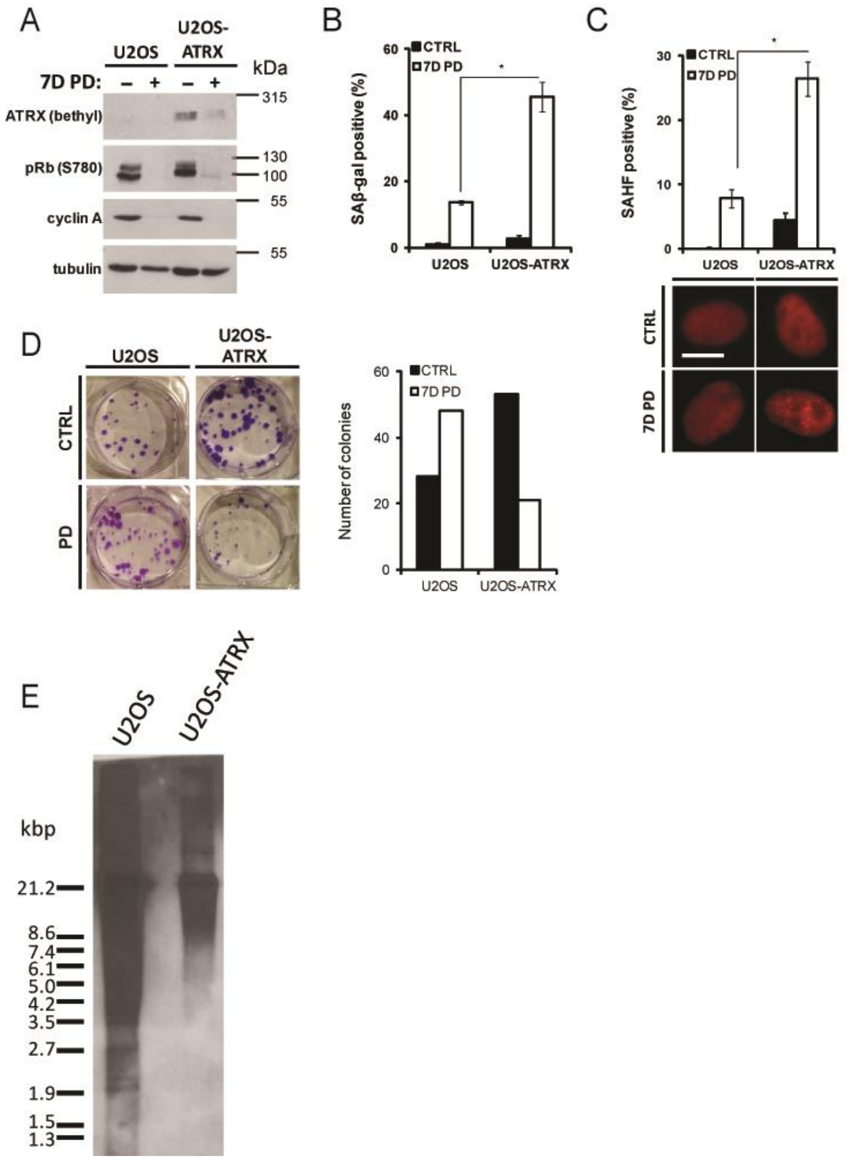

Supplementary Figure 3. ATRX facilitates senescence in response to CDK4 inhibition in the ATRX-deficient cell line U2OS. U2OS cells were stably transfected with wild type ATRX as described in the legend to Figure 3. **(A)** The expression of cyclin A and phosphorylated Rb before and after drug treatment in parental U2OS cells and U2OS cells expressing ATRX was determined by immunoblot. **(B-C)** The accumulation of SA-β-gal (B) and SAHF (C) were measured in each condition. Representative SAHF images are shown in panel C. **(D)** Clonogenic growth was measured by crystal violet staining three weeks after the removal of the drug and replating the cells at low density in the absence of drug. Representative images are shown (left) and colony number is quantified (right). **(E)** Telomere restriction fragment length assay was performed on 1μg of genomic DNA in the indicated cell lines. All data are represented as mean ± SEM from at least 3 independent experiments. * indicates $p < 0.05$ using a two-sided Students *t*-test. Scale bar indicates 20 microns.

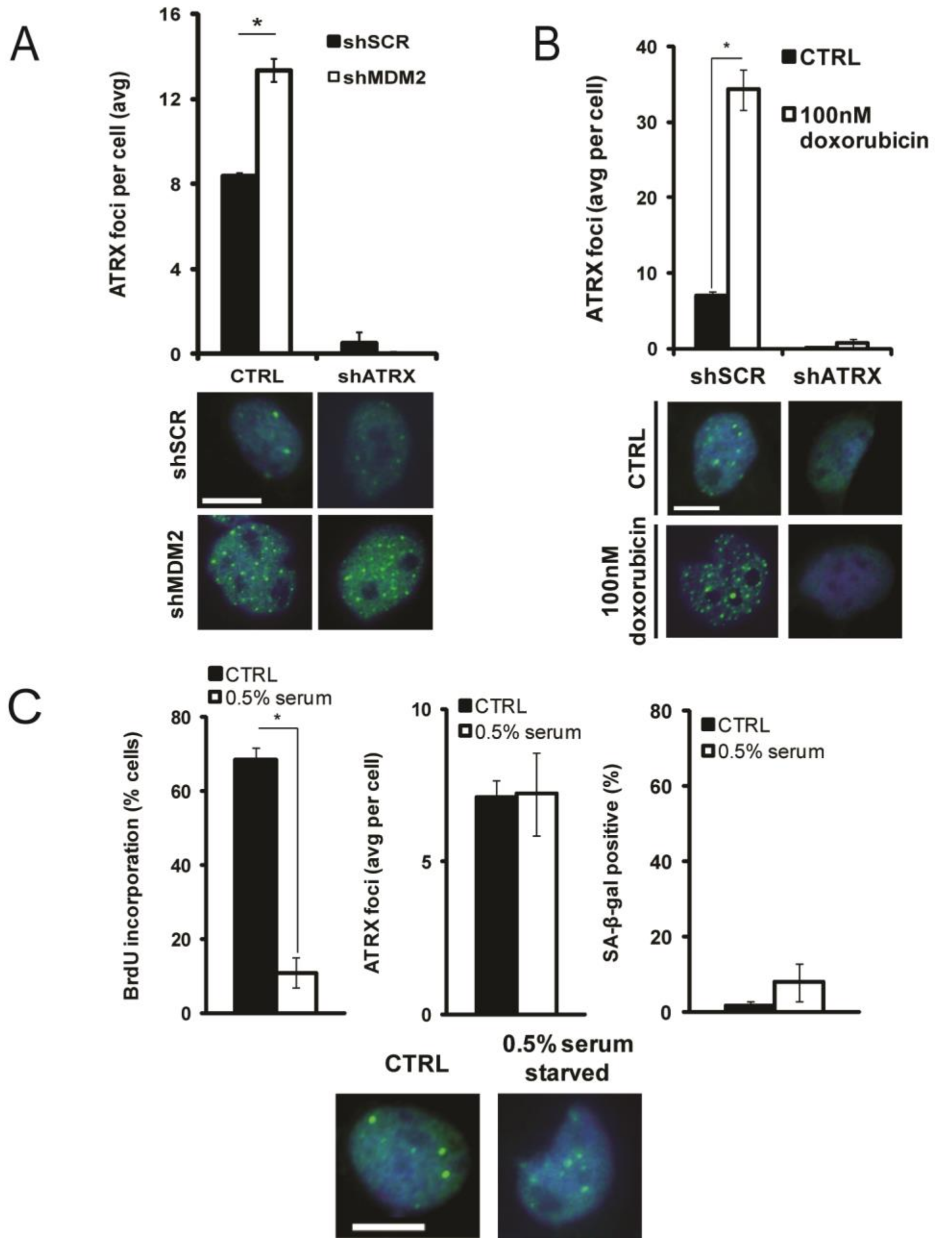

Supplementary Figure 4. The number of ATRX foci increases in senescent but not quiescent LS8817 cells. (A) ATRX immunofluorescence was carried out in LS8817 cells in which senescence was induced by MDM2 knockdown as described in the legend to Supplementary Figure 2. Representative images are shown below. **(B)** $LS8817^{scr}$ and $LS8817^{shATRX}$ were treated with doxorubicin as described in the legend to Figure 1 and ATRX foci detected by immunofluorescence, counted, and average number plotted. Representative images are shown below. **(C)** LS8817 cells were serum starved (0.5%) for 5 days. The proliferation of these cells was assessed by BrdU incorporation. Senescence was assessed by the accumulation of SA-β-gal positive cells. The number of ATRX foci as measured as described previously. All data are represented as mean ± SEM from at least 3 independent experiments. * indicates $p < 0.05$ using a two-sided Students *t*-test. Scale bars indicate 20 microns.

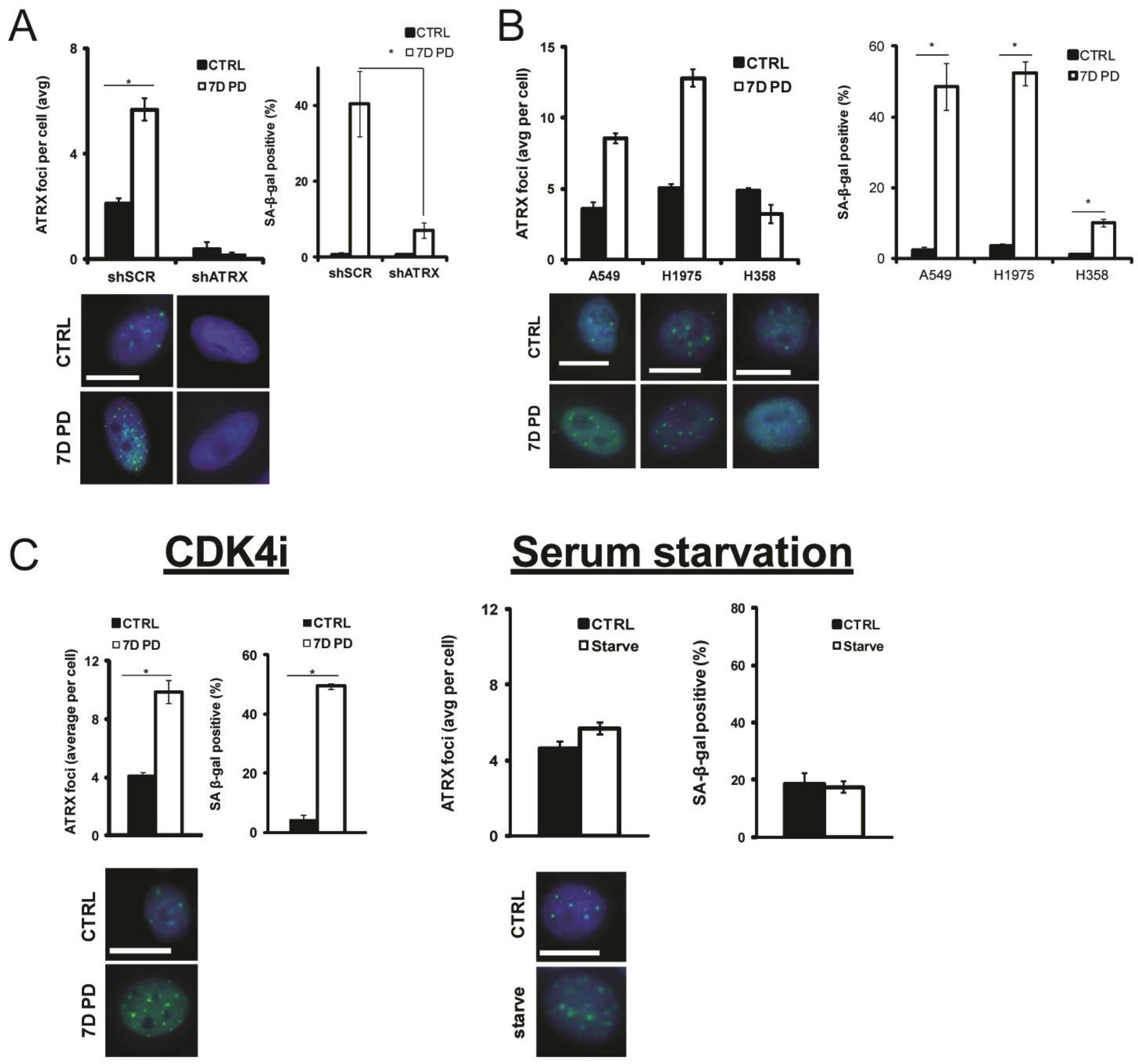

Supplementary Figure 5. ATRX foci accumulate in different transformed cells that undergo CDK4i induced senescence but not during quiescence. (A) SNB19 glioma cells were transduced with targeting vectors as described in the legend to Figure 1 and subsequently treated with PD0332991 and the accumulation of ATRX foci (left) and SA-β-gal positive cells (right) was measured. Representative images of ATRX foci are shown (bottom). **(B)** Three lung cancer derived cell lines were treated with PD0332991 for 7 days and the accumulation of ATRX foci (left) and SA-β-gal positive cells (right) was determined. Representative images of ATRX foci are shown (bottom). **(C)** MCF7 breast cancer cells were treated with PD0332991 for 7 days (left) or serum starved (0.5% serum) for 5 days (right). The accumulation of ATRX foci and SA-β-gal was measured as described in panel A. Representative images of ATRX foci are shown (bottom). All data are represented as mean ± SEM from at least 3 independent experiments. * indicates $p < 0.05$ using a two-sided Students *t*-test. Scale bars indicate 20 microns.

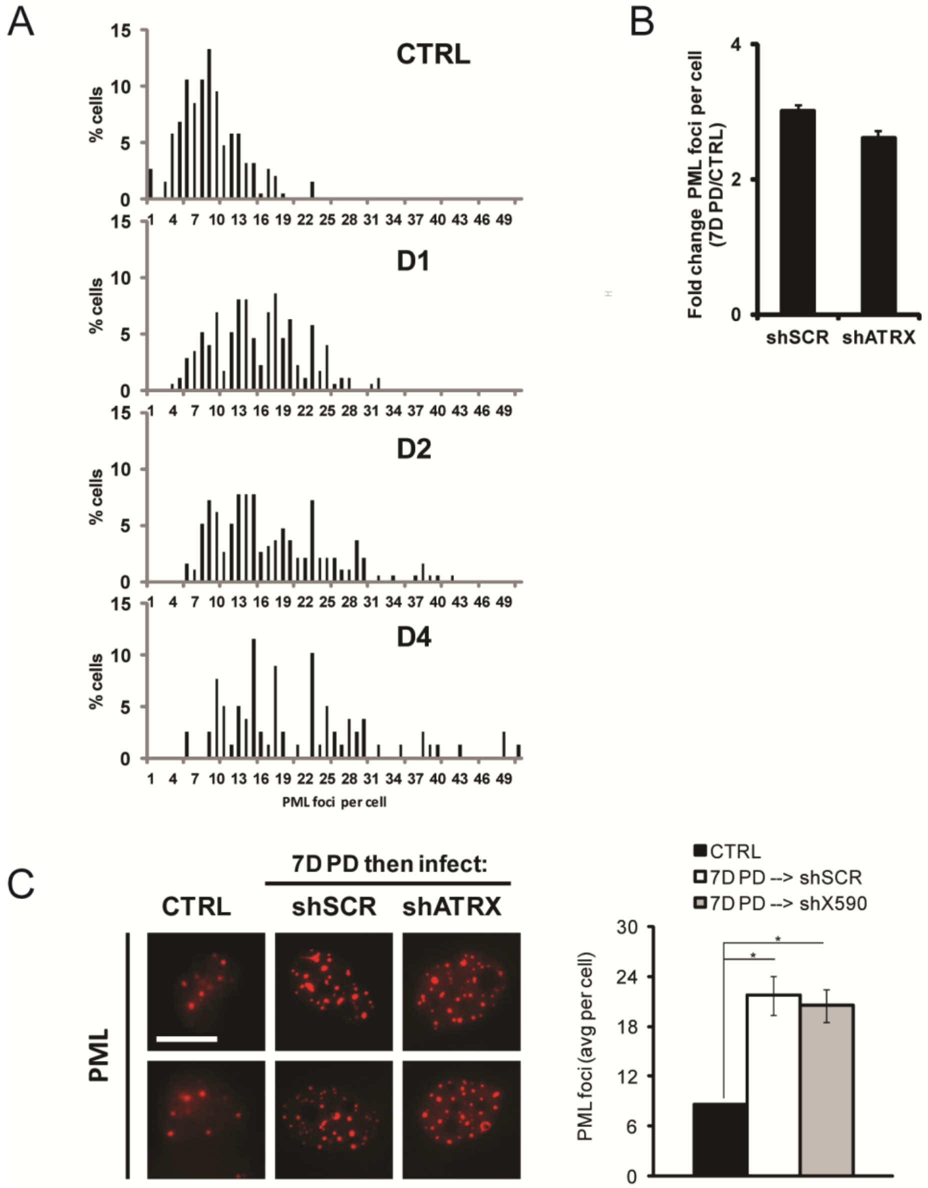

Supplementary Figure 6. Loss of ATRX does not affect PML foci. (A) PML foci were stained in LS8817 cells treated with CDK4 inhibitor and the number of foci was plotted as described in the legend to Figure 2A. **(B)** The presence of PML foci was assessed in LS8817^{scr} and LS8817^{shATRX} cells after treatment with PD0332991 for seven days. Graph represents the fold-change in the number of foci per cell in PD0332991 treated cells vs. untreated controls. **(C)** LS8817 cells were treated as described in the diagram in Figure 4A. PML foci were detected and quantified as described previously. Representative images are shown (left) and the number of PML foci per cell is quantified (right All data are represented as mean ± SEM from at least 3 independent experiments. * indicates $p < 0.05$ using a two-sided Students *t*-test. Scale bar indicates 20 microns.

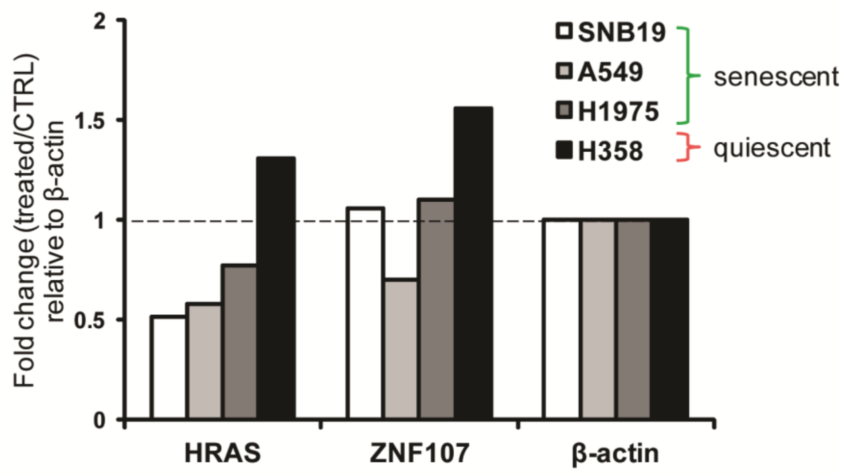

Supplementary Figure 7. *HRAS* is repressed in a variety of different transformed cell lines undergoing senescence. The cells were treated as indicated and expression of *HRAS*, *ZNF107*, and *β-ACTIN* were measured as described. The outcome of treatment, vis a vis quiescence or senescence is indicated in the graph.

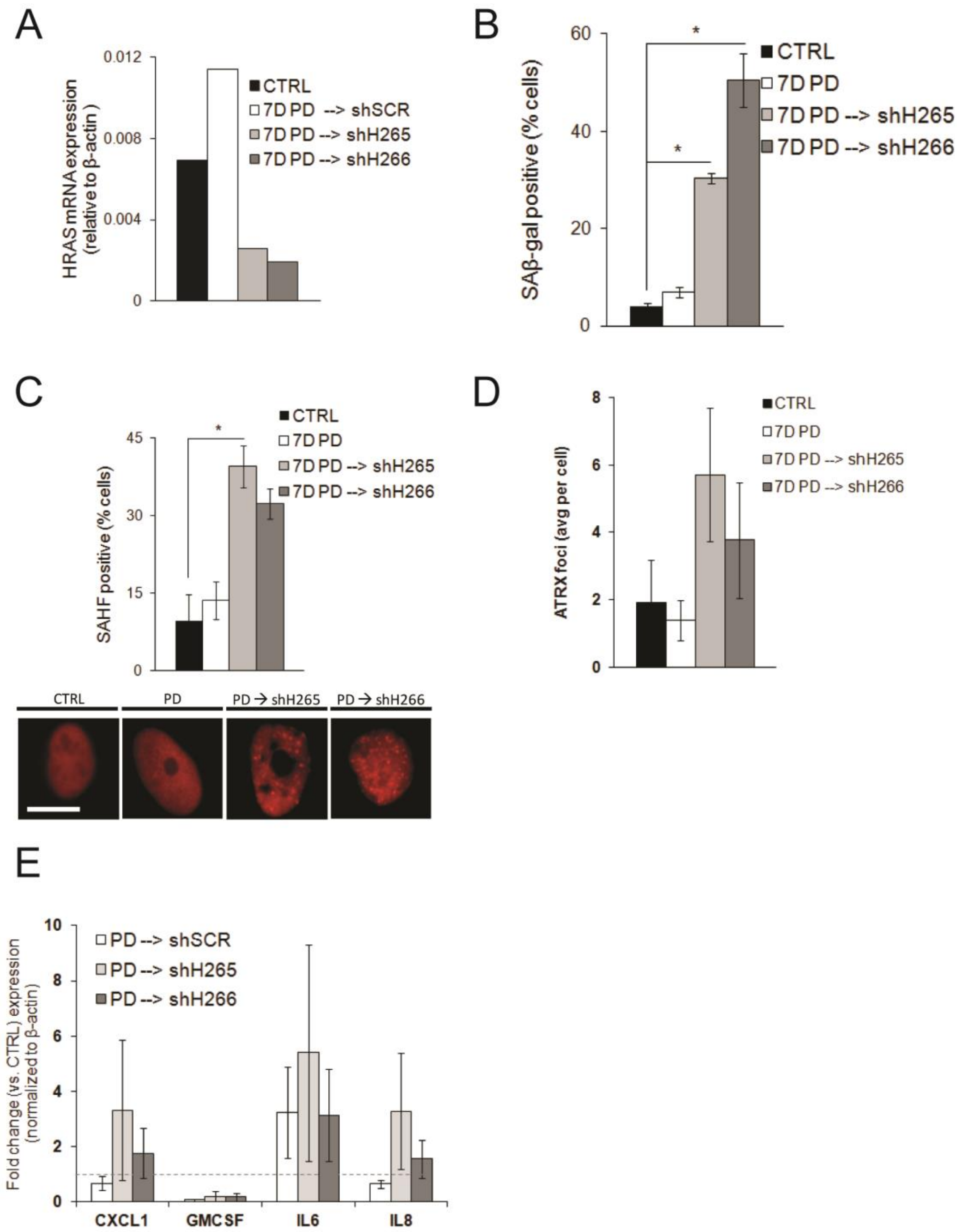

Supplementary Figure 8. Altering the level of *HRAS* expression regulates the transition from quiescence to senescence. LS8107 cells were treated with PD0332991 for seven days and then infected with lentiviral vectors targeting *HRAS*. PD treatment was maintained as viral selection occurred. **(A)** The efficacy of knockdown was assessed by qPCR. **(B-E)** The effect of reducing *HRAS* on the accumulation of SA-β-gal (B), SAHF (C), or ATRX foci (D) and the induction of SASP mRNAs as measured by qPCR (E) was determined as described. Representative SAHF images are shown in panel C. All data are represented as mean ± SEM from at least 3 independent experiments. * indicates $p < 0.05$ using a two-sided Students *t*-test. Scale bar indicates 20 microns.

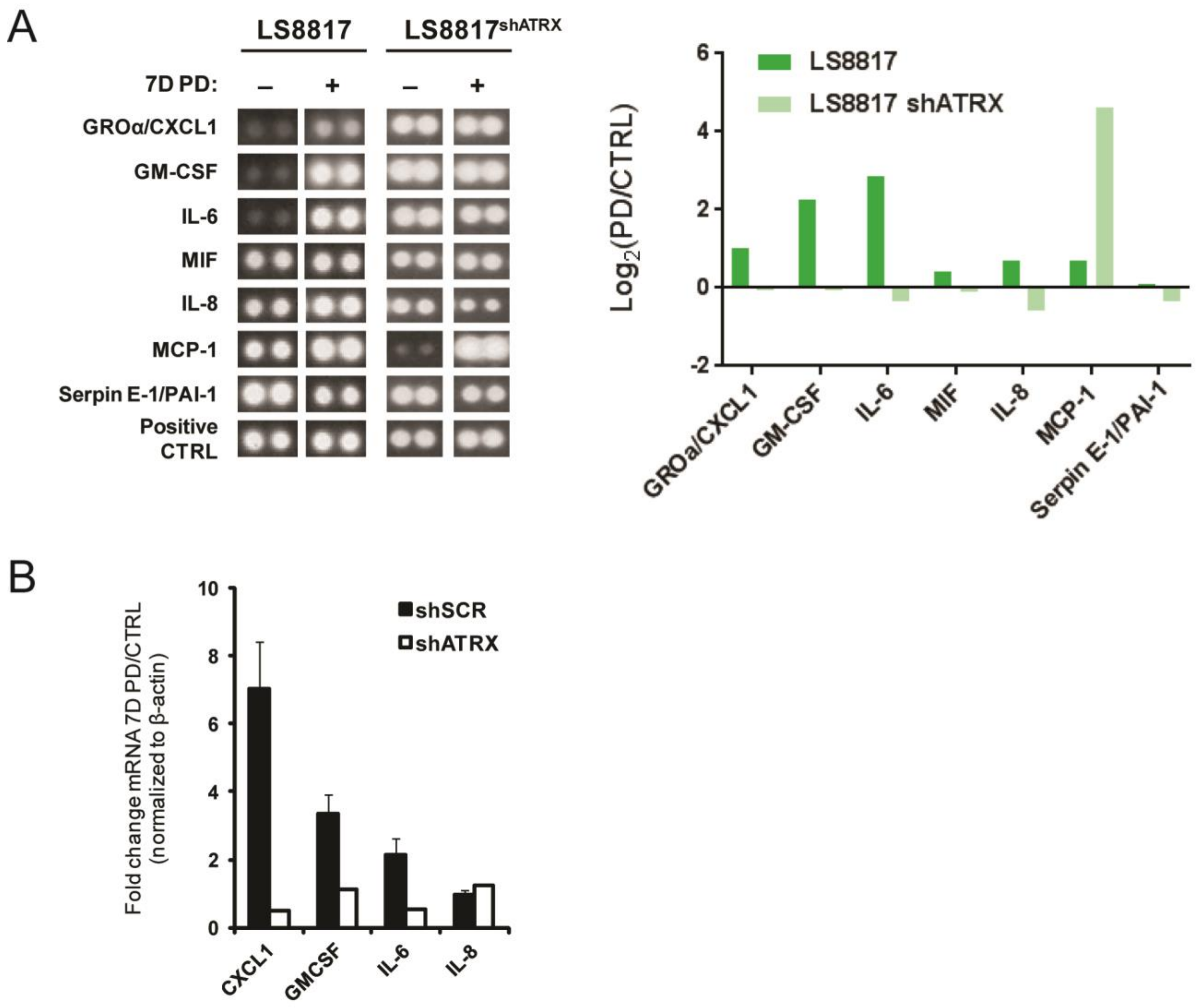

Supplementary Figure 9. Reducing ATRX suppresses the SASP in response to CDK4 inhibition in LS8817 cells. ATRX was reduced in LS8817 cells as described in the legend to Figure 1 and cells were treated with PD0332991 for seven days (7D PD). **(A)** Conditioned media was collected and cytokine expression measured as described in the methods. Representative images are shown (left) and induction of secreted SASP factors is shown (right). **(B)** mRNA was harvested from the indicated LS8817 cells as described in the methods and the induction of expression of the indicated SASP factors was measured.

Figure 1A

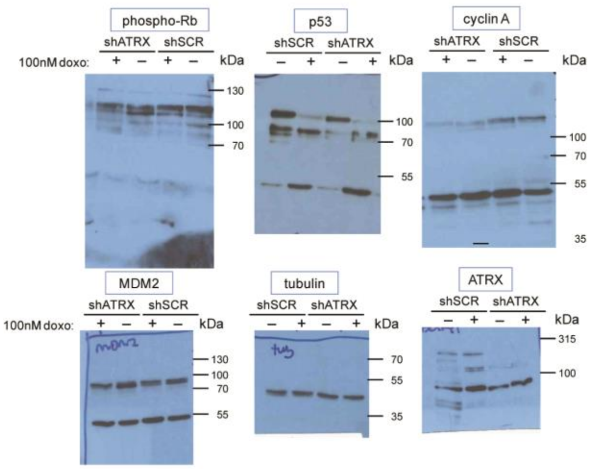

Figure 3D

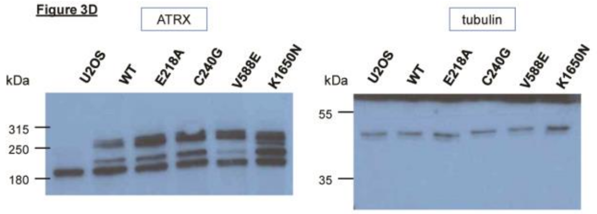

Figure 3E

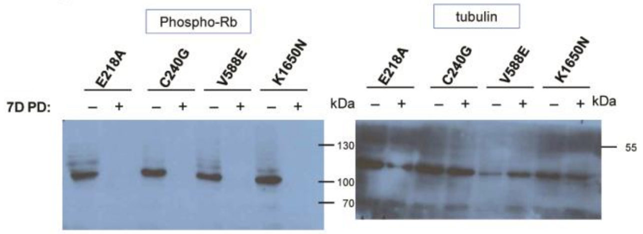

Figure 7C

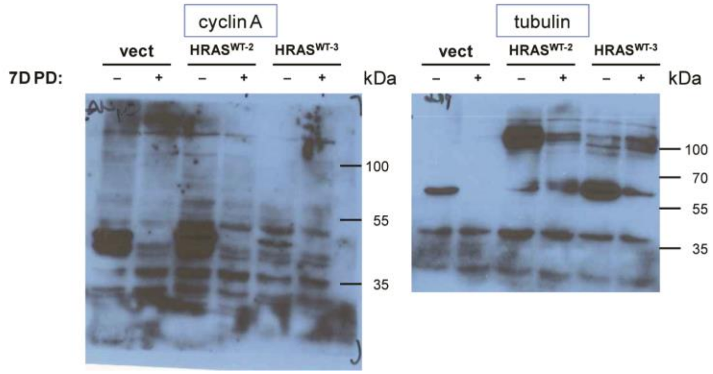

Supplementary Figure 10. Uncropped immunoblots. Blots from the corresponding figures were probed for the protein as indicated above.

Supplementary Table 1. Intergenic loci of senescence-specific ATRX peaks.

Chrom	start	end	Annotation
**1**	91925938	91925939	
	102018219	102018404	
	105412514	105412523	
	209408416	209408486	
	239388803	239389186	
	249240657	249240661	
**2**	13501361	13501428	
	18476404	18476451	
	68677257	68677357	
	89830549	89830948	Satellite repeat
	89842097	89842579	Satellite repeat
	90372669	90373039	Satellite repeat
	170324275	170324747	
	234696198	234696415	
**3**	5280825	5281398	
	176015569	176015636	
**4**	3783531	3783536	
	12668659	12668821	
	32841093	32841720	
	49093261	49093513	Centromeric repeat
	49101046	49101397	Centromeric repeat
	49152326	49152682	Centromeric repeat
	49644961	49645328	Centromeric repeat
	72550758	72550863	
	174406163	174406321	
	1871481	1871550	
**5**	18762886	18763065	
	10347767	10347851	
	10347928	10347944	
	10861184	10861241	
	49941286	49941585	
	55302722	55303293	
	97567854	97567952	
	123085501	123085540	
	152076921	152077079	
	152077575	152077644	
**6**	12167595	12167898	
	28557300	28557702	
	95950978	95950985	
	115389236	115389304	
	115389604	115389811	
	156028585	156028662	
**7**	303448	303662	
	13410402	13410825	
	1368078	1368166	
	24470223	24470403	
	61753367	61753692	Centromeric repeat
	61754739	61754758	Centromeric repeat
	61755180	61755181	Centromeric repeat
	61785764	61785943	Centromeric repeat
	61976292	61976485	Centromeric repeat
	64686706	64686764	
	64687462	64687553	
	64688394	64688449	
	67957852	67957855	
	89587945	89588409	
	109256914	109256962	
	112436507	112436597	
	112437364	112437777	
	152595074	152595085	
**8**	580611	581274	
	123511634	123511942	
	144830890	144831040	
**10**	4538086	4538309	
	35212938	35214343	
	35273735	35274491	

	36579319	36579973	
	38817263	38817894	Satellite repeat
	42376312	42376513	Centromeric repeat
	124406499	124406826	
	124845541	124845586	
**12**	28802780	28803520	
	90486997	90487035	
**13**	45998983	45999069	
**14**	19349311	19349576	
**15**	68132281	68132735	
**16**	14379872	14379886	
	46385813	46386377	Satellite repeat
	46401210	46401342	Satellite repeat
	46418455	46418660	Satellite repeat
	46419752	46420071	Satellite repeat
	46425345	46425933	Satellite repeat
	46427376	46427635	Satellite repeat
	46427947	46428131	Satellite repeat
	46437417	46437826	Satellite repeat
	46438756	46439060	Satellite repeat
	46453996	46454222	Satellite repeat
**17**	1107027	1107069	
	25265249	25265420	Satellite repeat
	77868506	77868574	
**18**	14689273	14689486	
	23044031	23044157	
	28338370	28338473	
	28338647	28338876	
	74220747	74220815	
**19**	246216	246233	TAR1
**22**	18715549	18715619	
	18716885	18717001	
	18719183	18719205	
**X**	25695192	25695281	
	25695736	25695898	
	25696006	25696064	
	26102926	26102957	
	26103019	26103701	
	26134399	26135146	
	58073171	58073271	
	61724028	61724201	Centromeric repeat
	73765939	73765967	
	77524597	77524999	

The senescence-specific summits (of the 166) that were not associated with either gene bodies or promoter regions are listed by chromosomal location. Annotated ENCODE blacklist regions within the locus are noted.

Supplementary Table 2. List of primers used for DNA sequencing and qPCR

	Forward Primer	Reverse Primer
ARHGAP18	5'-ATCAAGAGGTGGTTGTTGTCAAA-3'	5'-ACAATGCTTTCCTGTGGATCTC-3'
ATAD2	5'-GGAAAAACCTCGTCACCAGAG-3'	5'-CGCCTGTTCATTCGTTTACAGTA-3'
CCNY	5'-CAGACGGACGTGAGAGAAAAA-3'	5'-TGGTTGACTGACTGTGCTATCA-3'
DHX9	5'-GCAGCAGAGTGTAACATCGTAG-3'	5'-ACTCAAATCGAACGCTGTAGC-3'
HRAS	5'-CAGATCAAACGGGTGAAGGAC-3'	5'-GCCTGCCGAGATTCCACAG-3'
KIF15	5'-CTGAAGCCTATCAGGTGTTGTC-3'	5'-AGGGAGGTCCGTATATTCACAAT-3'
PARD3	5'-GCCTTTCAGCCTTACCAAGCA-3'	5'-GGTCACTACTGCGTCGAACAT-3'
SORCS3	5'-GAATGTGCCGAGACAACTAGAA-3'	5'-AGACACGTAGTAGCTGGCTCT-3'
ZNF107	5'-TTTTCATTTTGCCCAAGACC-3'	5'-GCTTGGGGTAGCTGTCAAAC-3'
NRAS	5'-TGATTTGCCAACAAGGACAG-3'	5'-CCCTGAGTCCCATCATCACT-3'
KRAS	5'-TACAGTGCAATGAGGGACCA-3'	5'-TCCTGAGCCTGTTTTGTGTCT-3'
CTNND2	5'-GCCGGAAGTGATTCAGATGT-3'	5'-CTACGGTGGACTTCGGTCAT-3'
HCN1	5'-CCAGCTGATATGCGTCAGAA-3'	5'-GGCATTGTAGCCACCAGTTT-3'
PCLB1	5'-CCTCGTGAACATCTCCCATT-3'	5'-GCTTCCGATCTGCTGAAAAC-3'
RNF128	5'-ATGATCGGCAATCTGAAAGG-3'	5'-GCTTTGAGCTCTTGCATTCC-3'
ATRX	5'-GTAGACAAGCCAGCCAGGAG-3'	5'-CATGAGGTGACCCAGTGTTG-3'
C20ORF195	5'-CCCTGCGTGAGTTCCTGAC-3'	5'-CCTCGATGTAGTAGGAGCACTTA-3'
COL26A1	5'-GGCACTCTGTCTTCACAGCA-3'	5'-TGGGGAGAGCACCTTTATTG-3'
DGKB	5'-GTTGCAGAATACCTTGAGTGGG-3'	5'-CCAGAGACACGGTTCCATCAT-3'
GAS6-AS1	5'-CGTCGATGTGGTCATAGCAG-3'	5'-CTGAGGAGGTCAGGTTCTGG-3'
HIBADH	5'-TGCTGCCCACCAGTATCAATG-3'	5'-GCAGGATCAATAGTGCTGGAATC-3'
HRH1	5'-CTGAGCACTATCTGCTTGGTC-3'	5'-AGGATGTTCATAGGCATGACGA-3'
SIN3B	5'-AAGACGCCCTCACCTATCTG-3'	5'-GGGAAGAAAAGCGTTGAATCCAA-3'
TRPS1	5'-GACTGCATAACCGCACCAG-3'	5'-CCTGCACATCATAGGTCCCATT-3'
IL6	5'-ACTCACCTCTTCAGAACGAATTG-3'	5'-CCATCTTTGGAAGGTTCAGGTTG-3'
IL8	5'-ACTGAGAGTGATTGAGAGTGGAC-3'	5'-AACCCTCTGCACCCAGTTTTC-3'
GMCSF	5'-TCCTGAACCTGAGTAGAGACAC-3'	5'-TGCTGCTTGTAGTGGCTGG-3'
CXCL1	5'-AGGGAATTCACCCCAAGAAC-3'	5'-TGTTCAGCATCTTTTCGATGA-3'
CEBPα	5'-CACCTGCAGTTCCAGATCG-3'	5'-CCGGTACTCGTTGCTGTTCT-3'
PPARγ	5'-ACCAAAGTGCAATCAAAGTGGA-3'	5'-ATGAGGGAGTTGGAAGGCTCT-3'
FABP4	5'-ACTGGGCCAGGAATTTGACG-3'	5'-CTCGTGGAAGTGACGCCTT-3'
ATRX ADD DOMAIN	5'-CAGAGCTTGCCAAAAGATGG-3'	5'-CCAATGCAAGATGAGCCTTC-3'
ATRX HP1 BINDING DOMAIN	5'-GCAGCAGTGGAACTGAACAA-3'	5'-TTTCTGTCGGTCGCCTCA-3'
ATRX HELICASE 1 DOMAIN	5'-AAGATGCTTCACCCACCAAG-3'	5'-GCTGCAAACACCAAGGATGA-3'
ATRX HELICASE 2 DOMAIN	5'-CAGATGCTGATGCTGAGGTT-3'	5'-TGGTCCCAGTTGGTATGTTG-3'
HRAS	5'-TCGTCCACAAAATGGTTCTG-3'	5'-TGAGGAGCGATGACGGAATA-3'
ZNF107	5'-CCAACACTCAAACCTAATTAACCA-3'	5'-GGTTTGAAGACTGGCTAAAAGC-3'

mRNA expression

sequence confirmation

ChIP-qPCR
